# Self-Assembly of Chiral Cyclohexanohemicucurbit[*n*]urils with Bis(Zn Porphyrin): Size, Shape, and Time-Dependent Binding

**DOI:** 10.3390/molecules27030937

**Published:** 2022-01-29

**Authors:** Marko Šakarašvili, Lukas Ustrnul, Elina Suut, Jagadeesh Varma Nallaparaju, Kamini A. Mishra, Nele Konrad, Jasper Adamson, Victor Borovkov, Riina Aav

**Affiliations:** 1 Department of Chemistry and Biotechnology, School of Science, Tallinn University of Technology, 12618 Tallinn, Estonia; marko.sakarasvili@taltech.ee (M.Š.); lukas.ustrnul@taltech.ee (L.U.); elina.suut@taltech.ee (E.S.); jagadeesh.varma@taltech.ee (J.V.N.); kamini.mishra@taltech.ee (K.A.M.); nele.konrad@taltech.ee (N.K.); 2 Laboratory of Chemical Physics, National Institute of Chemical Physics and Biophysics, 12618 Tallinn, Estonia; jasper.adamson@kbfi.ee

**Keywords:** supramolecular chemistry, noncovalent interaction, hemicucurbituril, bis–porphyrin, metalloporphyrin, chirogenesis, induced chirality, chiral recognition, circular dichroism, self-assembly, sensing

## Abstract

In order to investigate the ability of bis(zinc octaethylporphyrin) (**bis–ZnOEP**) to discriminate cyclohexanohemicucurbit[n]urils (**cycHC[*n*]**) of different shapes and sizes, the self-assembly of barrel-shaped chiral **cycHC[*n*]** with **bis–ZnOEP** was studied by various spectroscopic methods (absorption, fluorescence, circular dichroism (CD), and NMR). While the binding of 6-membered **cycHC[6]** induced a tweezer-like conformation followed by the formation of *anti-*form of **bis–ZnOEP** upon further addition of **cycHC[6]**, the interaction of 8-membered **cycHC[8]** is more complex and proceeds through the featured *syn*-to-*anti* conformational change of **bis–ZnOEP** and further intermolecular self-assembly via multiple noncovalent associations between **cycHC[8]** and **bis–ZnOEP**. Whilst bis–porphyrins are known to be effective chemical sensors able to differentiate various guests based on their chirality via induced CD, their ability to sense small differences in the shape and size of relatively large macrocycles, such as chiral **cycHC[6]** and **cycHC[8]**, is scarcely examined. Both studied complexes exhibited characteristic induced CD signals in the region of porphyrin absorption upon complexation.

## 1. Introduction

Porphyrins and their derivatives are one of the cornerstones of life; they are responsible for storing and transporting oxygen [1], photosynthesis [2], and enzyme-catalyzed reactions [3,4]. Synthetic derivatives have a wide range of applications, e.g., catalysis, photodynamic therapy, and sensing [5,6,7,8,9]. The versatility of porphyrin application can be significantly improved by metal insertion and/or covalently linking two porphyrin rings into a bis–porphyrin structure. The corresponding linker(s) may provide conformational flexibility or rigidity and thus make it possible to vary the distance between corresponding cores [10,11,12,13,14,15,16]. The most exciting aspect of bis–metalloporphyrins is their ability to recognize enantiomers of various monodentate and bidentate chiral molecules [11,17,18,19,20,21,22] related to the general phenomenon of chiral information transfer from guest to host, which is also employed by other sensing systems [23,24,25,26]. Typically, upon the host–guest interaction, a noticeably strong circular dichroism (CD) signal in the region of porphyrin absorption is induced. This is because the bis–porphyrin molecule must accommodate its conformation to the guest’s chiral structure, hence resulting in chirality transfer from a chiral guest to achiral bis–porphyrin [18]. Moreover, the intensity of the signal can be further enhanced by exciton coupling (EC) caused by the through space interaction between corresponding electronic transitions of a bis–porphyrin [10,27]. As a result, bis–metalloporphyrins can be sensitive not only to the stereochemistry of a guest but also to the bulkiness and shape of its substituents. Such properties were well described on the widely studied bis–porphyrin structure with a simple flexible ethane link between two porphyrin rings: bis(zinc(II) octaethylporphyrin) (**bis–ZnOEP**) [18,28,29].

**Figure 1:**
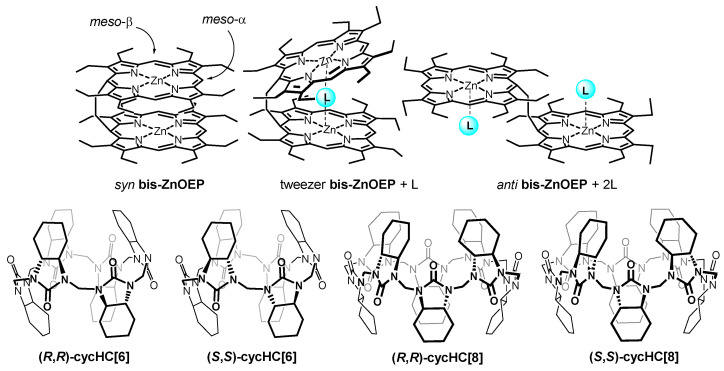
On top: structure of **bis–ZnOEP** in *syn*, tweezer, and *anti* conformations, and beneath: structures of (*R*,*R*)- and (*S*,*S*)-**cycHC[*n*]**.

In a noncoordinating solvent, **bis–ZnOEP** favors a remarkably stable *syn* face-to-face conformation (Figure 1) due to strong π–π interactions between the porphyrin rings [18]. Upon the binding of an external guest, the π–π interactions are disrupted, and **bis–ZnOEP** can be rearranged into two major conformations: (1) tweezer-like conformation with a guest positioned between two porphyrin rings, a conformation especially favored in the complexes with bidentate guests, and (2) opened *anti* conformation with two porphyrin rings separated from each other in an almost parallel arrangement, a conformation typical for complexation with monodentate guests. If the guest is chiral, induced CD (ICD) can be observed with an intensity related to EC. In turn, EC exhibits a parabola-like dependence on the dihedral angle between the porphyrin cores with no EC for the dihedral angles of 0° and 180° [28,30,31,32]. The tweezer conformation with a chiral guest conventionally provides strong EC and intense ICD, due to the exceptional rigidity and unidirectional helicity of the formed supramolecular complex. However, in the case of a more flexible *anti* conformation, ICD may strongly depend upon the corresponding complex geometry, which can be parallel (no EC) or slightly distorted to form a screw structure (relatively weak EC) [31,33,34,35].

Recently, we reported that carbonyl groups of (*R*,*R*)- and (*S*,*S*)-enantiomers of barrel-shaped macrocycles, cyclohexanohemicucurbit[*n*]urils (**cycHC[*n*]**, *n* = 6, 8) (Figure 1), can externally bind multiple zinc porphyrins through the Lewis acid-base interactions and subsequently induce chirality at the porphyrin core [36]. A family of **cycHC[*n*]**s consists of chiral and nonchiral (*n* = 6, 8, 12) members [37,38,39,40] and features, analogous to all single bridged cucurbiturils [41,42], binding anions inside the macrocycle cavity. Additionally, **cycHC[*n*]**s bind hydrogen bond donors and electron-rich organic molecules [43,44]. Upon complexation of chiral **cycHC[*n*]**s with achiral and CD silent zinc octaethylporphyrin (**ZnOEP**) and zinc tetraphenylporphyrin (**ZnTPP**) in CH_2_Cl_2_, an ICD signal is observed in the region of the porphyrin Soret band [36]. This inspired us to explore further complexation and chirogenesis with **bis–ZnOEP** due to its aforementioned binding and chirality sensing abilities. Moreover, the complexation of **bis–ZnOEP** with relatively large multidentate molecules, such as **cycHC[*n*]**s, has not yet been studied. Thus, we present a binding study employing various spectroscopic techniques (UV-vis absorption, fluorescence (FS), CD, NMR), including variable temperature (VT) and time-dependent measurements to characterize the corresponding supramolecular complexes of **bis–ZnOEP** with enantiomerically pure **cycHC[6]** and **cycHC[8]**, which have six and eight available urea binding sites, respectively.

## 2. Results and Discussion

### 2.1. Binding of ***Bis–ZnOEP*** with ***CycHC[6]*** and ***CycHC[8]***

**Figure 2:**
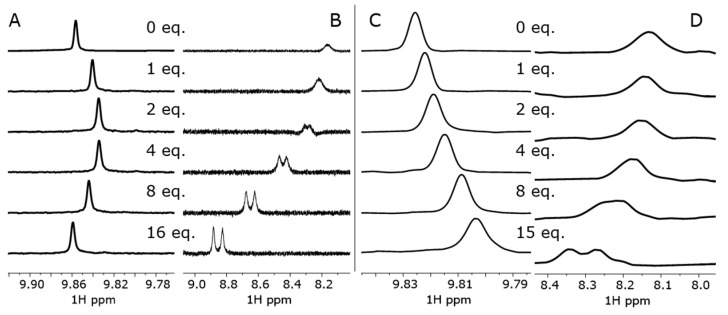
^1^H-NMR spectra of **bis–ZnOEP** upon addition of **cycHC[6]** in CD_2_Cl_2_: signals of (**A**) *meso*-α protons and (**B**) *meso*-β protons; and upon addition of **cycHC[8]** in CH_2_Cl_2_ and 10% CDCl_3_: signals of (**C**) *meso*-α and (**D**) *meso*-β protons.

The binding properties of **bis–ZnOEP** with **cycHC[*n*]**s were evaluated by ^1^H-NMR and UV-vis titrations using only (*R*,*R*)- enantiomers of chiral macrocycles. A stepwise addition of **cycHC[6]** to the *syn* form of **bis–ZnOEP** in CD_2_Cl_2_ shows the shielding of the *meso*-α protons of **bis–ZnOEP** (Figure 1 and Figure 2A). However, the addition of two equivalents of macrocycle was followed by the deshielding of the same signal in the presence of higher equivalency, which clearly indicates the presence of more than one structural transformation. The change in chemical shifts of signals is influenced by **cycHC[6]** binding and subsequent conformational switching of **bis–ZnOEP**. The conformational changes of **bis–ZnOEP** are generally evaluated by ^1^H-NMR signals from the *meso*-β protons (Figure 1 and Figure 2B). In *syn* conformation, the *meso*-β protons experience a strong ring-current effect from the neighboring porphyrin ring and, due to the symmetry-related magnetic equivalency, provide a shielded broad singlet. Upon binding an external guest, the geometry of **bis–ZnOEP** changes; the influence of the ring-current effect of the porphyrin core causes deshielding, and due to symmetry loss, the signal is split [29,45]. These trends were observed in the case of **cycHC[6]**, where chemical shifts of *meso*-β protons split and are deshielded by 0.69 ppm (Figure 2B), which clearly indicates that the **bis–ZnOEP** *syn* form opens upon complexation with **cycHC[6]**. Similar behavior was found for the complex formation of **cycHC[8]** with **bis–ZnOEP**, namely that the porphyrin *meso*-protons exhibited deshielding and splitting in the case of *meso*-β protons (Figure 2D) and shielding in the case of *meso*-α protons (Figure 2C). However, for **cycHC[8]**, the changes of chemical shifts were to a lesser extent, and the reverse deshielding of the chemical shift of the *meso*-α proton was not observed (Figure 2C).

Titration experiments revealed that the maximum observed shift of *meso*-β protons were 0.17 ppm with **cycHC[8]** and 0.69 ppm with **cycHC[6]**, whilst the signal of the *meso*-α proton shifted by 0.02 ppm in the presence of 15 equivalents of **cycHC[8]** and only 2 equivalents of **cycHC[6]**. Assuming that the extent of chemical shift change is caused by the abundance of complexed species, a seemingly weaker binding of **cycHC[8]** compared to that of **cycHC[6]** can be deduced. This is in line with our previously published study [36], wherein mono **ZnOEP** interacted with both **cycHC[*n*]**s through the same supramolecular mechanism; **cycHC[6]** was bound approximately five times stronger than **cycHC[8]**.

**Figure 3:**
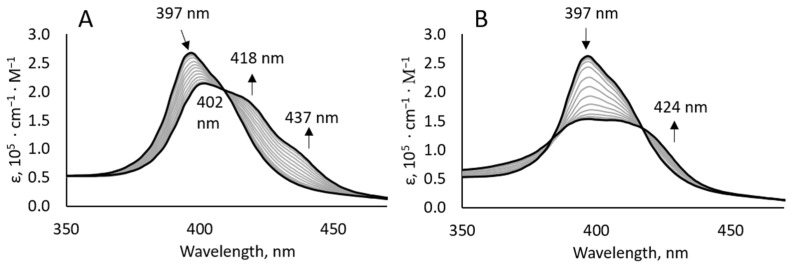
Absorption spectra of titration of **bis–ZnOEP** (3.2 × 10^−6^ M, CH_2_Cl_2_, 296 K) with (**A**) (*R*,*R*)-**cycHC[6]** from 0 to 2000 equivalents and with (**B**) (*R*,*R*)-**cycHC[8]** from 0 to 2000 equivalents.

The UV-vis titrations of **bis–ZnOEP** with **cycHC[6]** show bathochromic shifting of the porphyrin B band *λ*_max_ from 397 to 402 nm upon complexation. Additionally, further red-shifted absorptions appear at 418 and 437 nm (Figure 3A). These electronic transitions indicate the formation of a tweezer-like complex; they appear closely similar to the previously published complexation study for **bis–ZnOEP** with amino alcohols [35], where three well-resolved transitions were exhibited at 407 nm, as the main band, and at 418 and 435 nm, as corresponding shoulders.

Surprisingly, UV-vis titration of **bis–ZnOEP** with **cycHC[8]** showed a clear difference between the binding of two homologous macrocycles (Figure 3A,B). In particular, the complexation with **cycHC[8]** induced a decrease in the B band at *λ*_max_ 397 nm and appearance of the transition at 424 nm. This gave the absorption spectra of a contrastingly different shape at the same equivalents of **cycHC[8]**, as compared to **cycHC[6]**. Moreover, the saturation of the complex formation with **cycHC[8]** was seemingly reached at 1500 equivalents, while for **cycHC[6]**, more than 2000 equivalents were needed (Supplementary Materials pages S11–S15).

The ^1^H-NMR and UV-vis titration data of **bis–ZnOEP** with **cycHC[6]** were evaluated using the Bindfit online tool [46,47]. Only the 1:2 binding model gave a reasonable correlation between the experimental data and fitted the binding isotherm for both titration methods. The fitting of the ^1^H-NMR titration data (Supplementary Materials pages S3–S5) provided the corresponding association constants *K_1_* = 1650 ± 180 M^−1^ and *K_2_* = 183 ± 2 M^−1^, and the fitting of UV-vis titration data showed the binding strength of the same magnitude with *K_1_* = 4550 ± 470 M^−1^ and *K_2_* = 92 ± 1 M^−1^ (Supplementary Materials pages S11–S13). The association constants obtained by both methods clearly indicate a negative cooperativity of the binding process, meaning that the binding of the first **cycHC[6]** molecule diminishes the binding of the second one. Such behavior is also typical for **bis–ZnOEP** forming a 1:1 tweezer-like complex with bidentate guests [35]. However, an excess of the bidentate guest forces **bis–ZnOEP** to adopt a 1:2 *anti* conformation. Apparently, the second guest binding is less favorable due to additional energy needed to break interactions with the first guest molecule and also the conformational change of **bis–ZnOEP**. It should be noted that in the case of the guests that give 1:1 complex in *anti* conformation, the binding of the second guest has positive cooperativity. This is because the intramolecular interactions in **bis–ZnOEP** are destroyed, and the subsequent *syn*-to-*anti* conformational change occurs upon the binding of the first guest. Therefore, the second guest molecule can be freely bound to the second porphyrin core and has no additional constraints. Hence, as the negative cooperativity was observed, and on the basis of spectral data obtained, we can assume that the 1:1 complex with **cycHC[6]** has a tweezer-like conformation. Moreover, with *K_1_* in the 10^3^ M^−1^ range and *K_2_* being roughly 2–3 orders of magnitude smaller, the values correspond well with the previously described binding of smaller bidentate chiral guests, which formed the corresponding tweezer structure [35].

However, the evaluation of the ^1^H-NMR and UV-vis titration data for the **bis–ZnOEP** and **cycHC[8]** complex was unsuccessful with 1:2 binding models, and a reasonable fit was achieved only in the NMR concertation range (mM) for 1:1 binding with *K_1_* = 198 ± 5 M^−1^. Therefore, a different mechanism of the binding of **cycHC[8]**, as compared to **cycHC[6]**, should be considered. Additionally, certain time-dependent changes during the UV-vis titration experiments with **cycHC[8]** were noted.

### 2.2. Time-Dependent Behavior of Complexes

**Figure 4:**
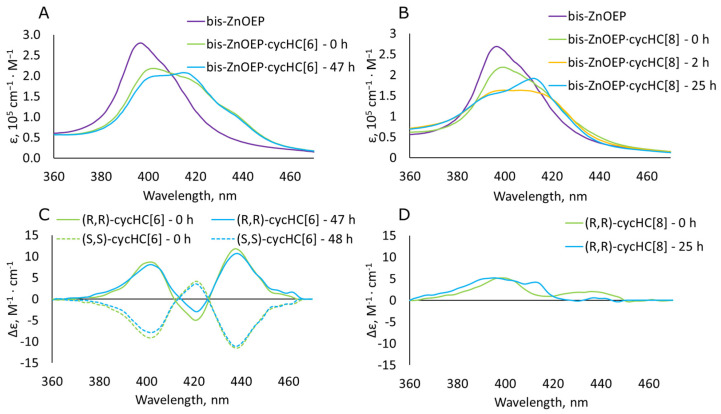
(**A**) Absorption spectra of free **bis–ZnOEP** (2.2 × 10^−6^ M, CH_2_Cl_2_, 296 K) and after single addition of 3000 eq of (*R*,*R*)-**cycHC[6]** in time. (**B**) Absorption spectra of free **bis–ZnOEP** (3.0 × 10^−6^ M, CH_2_Cl_2_, 296 K) and after single addition of 2200 eq of (*R*,*R*)-**cycHC[8]** in time. (**C**) Change of CD spectra in time corresponding to the sample of **bis–ZnOEP∙cycHC[6]** from absorption spectra A and for the complex with (*S*,*S*)-**cycHC[6]** measured at the same conditions. (**D**) Change of CD spectra in time corresponding to the sample of **bis–ZnOEP∙cycHC[8]** from absorption spectra B immediately after single addition and after 25 h.

The evaluation of the stability of **cycHC[*n*]** complexes in time was checked by measuring the UV-vis spectra of **bis–ZnOEP** immediately after the single addition of 2000 (or more) equivalents of **cycHC[8]** and **cycHC[6]** to provide sufficient abundance of the complex (see mole fractions on page S11) and compared with the data from the UV-vis titration first (pure **bis–ZnOEP)** and last points (complexed **bis–ZnOEP**) for the same macrocycles. The titration last points were measured approximately 1 h after the first addition and had the same equivalents of a guest as in the comparative single addition experiment (Figure 4A,B). In the case of **cycHC[6]**, the binding outcome was the same in both experiments (titration and single addition). As less pronounced changes in time were observed for the complex of **bis–ZnOEP** with **cycHC[6]**, we can conclude that the complex formation equilibrium is relatively fast within the measurement timeframe.

Conversely, in the case of the **cycHC[8]** complex, the distinct differences were spotted between the final spectrum of titration and single addition of the same equivalents of **cycHC[8]** (Figure 3B and Figure 4B, 0 h). The single addition (Figure 4B, 0 h) caused a lesser initial change in the spectrum, and noticeable spectral changes occurred in time (Figure 4B, 25 h), indicating a kinetically slow process. After 2 h, the spectra from a single addition experiment became similar to the last titration point (Figure 3B and Figure 4B, 2 h). After 24 h, further changes in the UV-vis spectra became negligible. Therefore, one can conclude that a relatively slow and concurrent kinetics of the complexation of **cycHC[8]** with **bis–ZnOEP** prevents the successful evaluation of association constants by the standard procedure.

**Figure 5:**
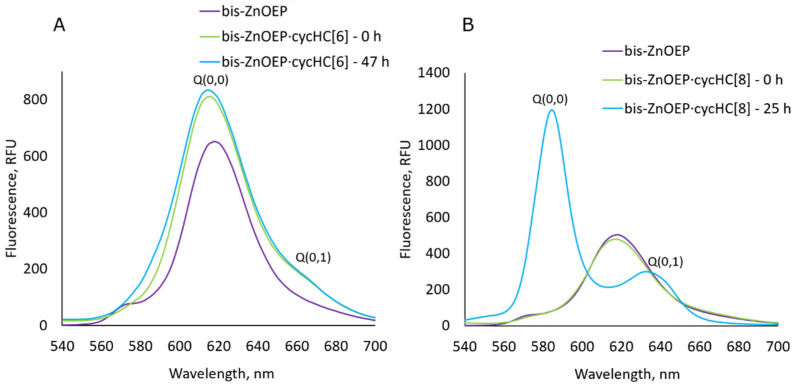
(**A**) Fluorescence spectra (*λ*_ex_ = 399 nm_)_ of free **bis–ZnOEP** (2.2 × 10^−6^ M, CH_2_Cl_2_, 296 K) and after single addition of 3000 eq of (*R*,*R*)-**cycHC[6]** in time (*λ*_ex_ = 404 nm_)_. (**B**) Fluorescence spectra of free **bis–ZnOEP** (3.0∙10^−6^ M, CH_2_Cl_2_, 296 K) and after single addition of 2200 eq of (*R*,*R*)-**cycHC[8]** in time (*λ*_ex_ = 413 nm).

To further study the kinetic process, additional CD and fluorescence spectra of **bis–ZnOEP** after a single addition of **cycHC[*n*]** were measured. In the case of **cycHC[6]**, minor changes in the spectra were noted. The ICD spectra (Figure 4C) exhibit a complex spectral profile consisting of three Cotton effects as a result of EC between two pairs of the porphyrin electronic transitions in **bis–ZnOEP**. The latter observation proves interaction with chiral **cycHC[6]** and corresponding helical distortion of **bis–ZnOEP**. The fluorescence spectrum in the presence of **cycHC[6]** showed the emissions of the complex at 611 nm, the Q(0,0) band, and at 660 nm, the Q(0,1) band (Figure 5A), which also support the formation of a tweezer-like complex as was suggested based on the UV absorption spectra. There are apparent similarities to the previously published complexes with small molecules that form tweezer-like structures [29].

Analogous to UV-vis data, a distinct complexation character of **bis–ZnOEP** with **cycHC[8]** was also observed in CD and fluorescence spectral data (Figure 4B,D and Figure 5B). The presence of the ICD signal of **bis–ZnOEP** upon mixing with **cycHC[8]** at the starting point (0 h) proves the formation of a chiral complex (Figure 4D). Although the shape of ICD is changed in time, all recorded spectra lack strong EC, resembling monosignate ICD of previously studied monomeric **ZnOEP** in complexes with **cycHC[*n*]** [36]. This along with the fact that the intensity of ICD remains low in time clearly indicates that chiral induction is apparently caused by unsymmetrical deformation of the individual porphyrin moieties at the nearly parallel-oriented porphyrin cores rather than by a unidirectional helical arrangement of the whole **bis–ZnOEP** molecule [48]. Moreover, a distinct contrast in the intensity and shape of the CD spectra of corresponding **cycHC[6]** and **cycHC[8]** complexes is additional evidence of the specific chiroptical selectivity of **bis–ZnOEP** as a chirality sensor for large chiral macrocycles (Figure 4C,D). Notably, further UV-vis kinetic experiments at higher concentration (3.4 × 10^−5^ M) of **bis–ZnOEP** also fully support this conclusion (see Supplementary Materials pages S16–S18 and S23).

Interestingly, the emission spectrum exhibited a negligible change directly after adding **cycHC[8]** to **bis–ZnOEP** (Figure 5B). However, the emission spectrum drastically changed after 25 h when the complex stabilized; it exhibited two distinct bands with the smaller Q(0,1) band at 632 nm and main Q(0,0) band at 585 nm of a higher intensity as compared to that at 0 h. These observations also suggest that the *syn*-to-*anti* conformational switching of **bis–ZnOEP** leads to fluorescence firing as the porphyrin cores become more spatially separated and hence less interactive. Similar changes have been also evidenced in previous studies [35]. This assumption is additionally supported by the similarity of the emission spectra shapes of monomeric **ZnOEP** and **bis–ZnOEP∙cycHC[8]** with the Q(0,0) and Q(0,1) bands of **ZnOEP** appearing at 577 and 628 nm, respectively (Figure 5B and Supplementary Material pages S24–S26). Moreover, upon the binding of **cycHC[8]**, the shape of the **ZnOEP** emission spectrum remained the same (Supplementary Material page S26).

The substantial change in time of **bis–ZnOEP∙cycHC[8]** UV-vis, CD, and FS spectra should arise either from slow *syn*-to-*anti* conformational change of **bis–ZnOEP** or aggregation of the host–guest complex. The time-dependent ^1^H-NMR measurements of the **bis–ZnOEP∙cycHC[8]** complex were performed in the 1:1 ratio and in excess of **cycHC[8]** to follow the conformational changes of **bis–ZnOEP** upon complexation. In addition, similar analysis of the **bis–ZnOEP∙cycHC[6]** complex was performed for comparison (see Supplementary Material page S28). Spectra were collected immediately after mixing and then 20 days later for solutions containing 1 equivalent of **cycHC[*n*]** and after 24 h for solutions containing excess of 8 equivalent of **cycHC[*n*]**. Time-dependent changes in the chemical shifts and signal shapes were not observed in neither of the complexes; however, the split in *meso-*β proton signals in excess of **cycHC[*n*]** clearly indicated opening of the *syn*-conformation of **bis–ZnOEP** in both **cycHC[*n*]** complexes (Figure 2 and Supplementary Material pages S3–S6 and S28) with comparably fast rate.

The observed difference in time-dependent behavior of the **bis–ZnOEP∙cycHC[8]** supramolecular system in the UV-vis, CD, and FS measurements, as compared to the NMR measurements, can be related to a substantial difference in the sample concentrations (μM and mM, respectively) and in excess of **cycHC[8]** (2000 and 8 equivalents, respectively). The low binding constant of **bis–ZnOEP** to **cycHC[8]** and relatively small excess of **cycHC[8]** in the NMR study might hinder the formation of the assembled species, as indicated by other methods. 

### 2.3. Variable Temperature ^1^H-NMR and Fluorescence Experiments

To find a process responsible for the time-dependent changes, two VT experiments were performed. 

**Figure 6:**
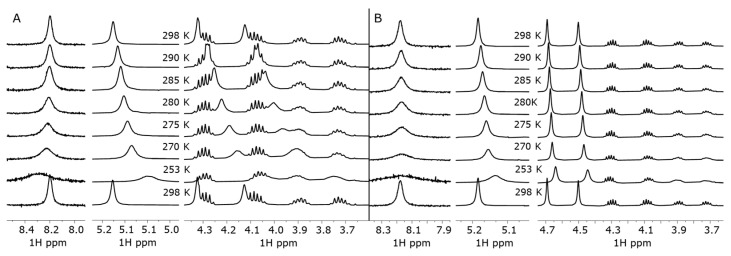
The selected areas of ^1^H-NMR spectrum of 1:1 ratio mixture of 1.0 × 10^−3^ M **bis-ZnOEP** and (**A**) (*R*,*R*)-**cycHC[6]** and (**B**) (*R*,*R*)-**cycHC[8]** at (from top to bottom): 298 (before cooling), 290, 285, 280, 275, 270, 253, and 298 K (after heating back to initial temperature).

Firstly, a stability and reversibility of the aggregate formation together with the thermodynamic parameters of the complex formation were studied by VT ^1^H-NMR of a samples containing 1:1 ratio of **bis–ZnOEP** and **cycHC[*n*]** (Figure 6 and Supplementary Materials pages S29–S30). Upon cooling down to 253 K, all the signals of **bis–ZnOEP** and both **cycHC[*n*]**s exhibited changes in chemical shifts, which can be related to the increased abundance of complexes due to a stronger binding at lower temperature. However, larger changes were observed in the presence of **cycHC[6]** complex in comparison to **cycHC[8]** (Figure 6A,B, respectively). The line broadening, caused by the slow exchange between complexed and noncomplexed species or aggregates, was most prominent in the shift of *meso-*β proton at 8.2 ppm in both samples; nevertheless, the coalescence point was not passed, and therefore, thermodynamic parameters could not be evaluated in this study.

Upon heating the samples from 253 back to 298 K, the observed changes were immediately reversed and resulted in the same ^1^H-NMR spectrum as observed prior to cooling (Figure 6). Therefore, the formation of new species that could be clearly identified as aggregates were not proven at mM concentration. 

**Figure 7:**
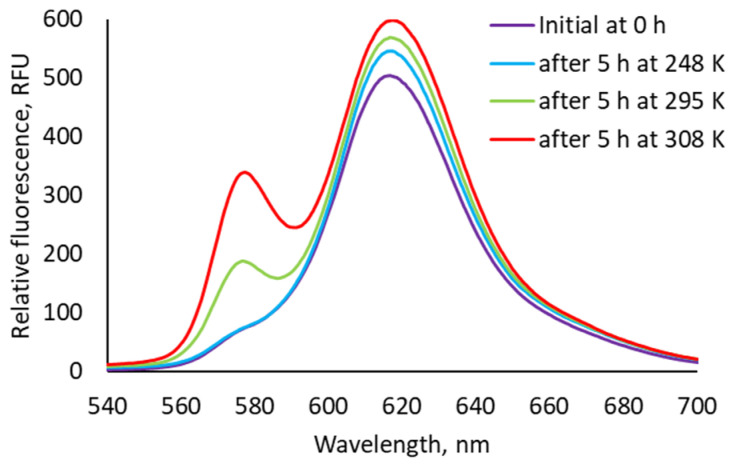
Emission spectra (excited at 397 nm) of **bis–ZnOEP** (3 × 10^−6^ M) samples with 2000 equivalents of **cycHC[8]** measured at 295 K immediately after preparation and then again after being kept for 5 h at different temperatures.

Secondly, the influence of temperature on the *syn*-to-*anti* conformational change was studied by fluorescence spectroscopy, as a method used for a µM concentration region. Samples containing mixtures of **bis–ZnOEP** and **cycHC[8]** were brought and kept at the temperature of 248, 295, and 308 K immediately after the samples preparation and then measured at 295 K (Figure 7). Based on the previous UV-vis experiments, it was estimated that maintaining the samples at different temperatures for 2–8 h should be sufficient to observe their differences; hence, samples were measured 5 h after the mixing of compounds. Importantly, the emission spectra were measured at 295 K; therefore, the observed differences can be attributed only to the time spent at different temperatures. The obtained results thus clearly show that at higher temperatures, the expected transformation of emission spectra progressed faster. This means that the conformational change from *syn*-to-*anti* form contributes in the kinetically slow process, stabilizing after approximately 24 h at room temperature. However, as noted above, the changes in emission spectra cannot be simply attributed only to the conformational change as opening of the *syn* conformation was relatively fast according to the NMR studies. Therefore, the aggregation of the complexes where **bis–ZnOEP** is in the *anti* conformation can be proposed at μM concentration range. 

### 2.4. Proposed Self-Assembly Mechanism

**Figure 8:**
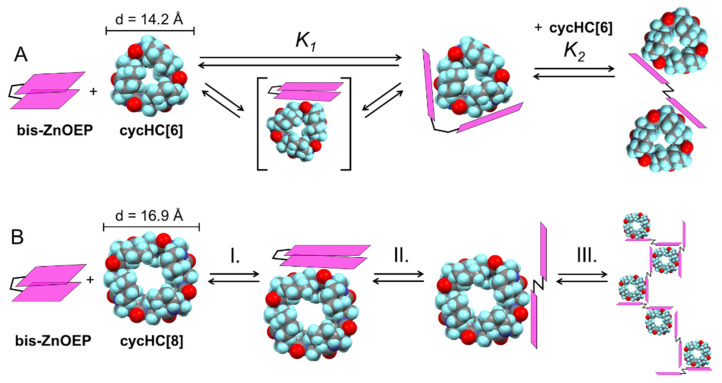
The proposed binding mechanisms of **bis–ZnOEP** with (**A**) **cycHC[6]** described by obtained association constants and with (**B**) **cycHC[8]**. The 3D structures and diameters of **cycHC[*n*]**s are based on previously reported crystal structures [36]. Color coding: C—gray, H—light blue, O—red, and N—dark blue.

The following binding and self-assembly mechanisms for the **bis–ZnOEP∙cycHC[*n*]** complexes were proposed based on the above-discussed experimental results (Figure 8). 

The interaction of **bis–ZnOEP** and **cycHC[6]** resembles the complexation of **bis–ZnOEP** with small bidentate guests [35]. Furthermore, the observed negative cooperativity is in line with the formation of a tweezer-like 1:1 complex, in which a molecule of **cycHC[6]** is placed between two porphyrin moieties and fixed by two coordination bonds. The further addition of **cycHC[6]** leads to the formation of 1:2 complex, which is in the *anti* form (Figure 8A). In the case of **cycHC[8]**, the self-assembly process is more complicated due to the presence of a slow kinetic process at µM concentration. Minor changes in absorption and fluorescence maximum wavelength and intensity shortly after mixing (Figure 4B and Figure 5B) suggest that the first binding (process I. at Figure 8B) is relatively weak and **bis–ZnOEP** is likely to preserve the *syn* conformation. Further time evolution in fluorescence proves the opening of **bis–ZnOEP** (II. and III.) via the kinetically slow process into the *anti* conformation, in which two porphyrin moieties are apart from each other, and as no EC was observed in CD, the angle between them is close to 180°. In addition, an aggregation (III.) of the complex would be the more probable explanation [49] for the slow process as opening of the *syn* conformation was proven to be fast by NMR studies. Nevertheless, *syn*-to-*anti* interconversion kinetics can be influenced by the concentration.

As both macrocycles are chemically analogous, the observed differences in binding mechanisms must be related to structural inequalities, such as bulkiness, shapes, and the number of binding sites. While the heights of **cycHC[6]** (12.4 Å) and **cycHC[8]** (11.9 Å) are similar, the diameters (d) and shapes obviously differ as a consequence of a differing number of monomers in **cycHC[*n*]** (Figure 8, Supplementary Material pages S31–S32). However, the diameter of **bis–ZnOEP** aromatic rings (12 Å) is comparable in size to measures of **cycHC[*n*]** (see Supplementary Material pages S31–S32). Clearly, a larger volume of **cycHC[8]** may lead to increased steric hindrance and preference for the opened *anti* conformation. Hence, it is reasonable to assume that the interaction between **bis–ZnOEP** and **cycHC[8]** is directed by the macrocycle size and steric hindrance caused by the ethyl groups of **bis–ZnOEP**.

Nevertheless, a probable relation between the guest’s bulkiness and kinetics of bis–porphyrin’s spectral changes at μM concentrations cannot explicitly explain why **cycHC[8]** could exhibit further aggregation while **cycHC[6]** does not. Although the binding of **cycHC[6]** proceeds through the tweezer-like complex—which is unsuitable for aggregation—an excess of both **cycHC[*n*]** leads to the final *anti* conformation, where both porphyrin rings of **bis–ZnOEP** are bound to the guests. This situation is essentially the same in the 1:2 *anti* complex and in the aggregate. Hence, the probable ability to aggregate with **cycHC[8]** but not with **cycHC[6]** can arise from the difference in shape and position of additional binding sites. As one can deduce from the ICD spectra, the specific geometry of **bis–ZnOEP·cycHC[8]** complexes is close to parallel, while **bis–ZnOEP·cycHC[6]** exhibits a helical distortion. Therefore, it is reasonable to assume that the geometry of one complex is suitable for aggregate formation, whereas the other is not. However, we are currently undertaking further study to elucidate the detailed structure of observed complexes, mechanism of self-association, and chirogenic processes and will report our findings in due course.

## 3. Materials and Methods

### 3.1. General

Unless otherwise stated, all reagents and solvents were purchased from commercial suppliers and used as received. Compounds prepared in our laboratories were **bis–ZnOEP** [50], (*S*,*S*) and (*R*,*R*)-**cycHC[8]** [38], (*S*,*S*) and (*R*,*R*)-**cycHC[6]** [37] and were prepared in all cases according to the procedures described in the literature. All solutions were prepared using Hamilton^®^ Gastight syringes (Hamilton Company, Reno, NV, USA); those syringes were also used for all additions during UV-VIS and NMR titrations. To ensure precise measurement in sample preparation, the mass of solvent and its density were used along with volumetric glassware. Samples were weighed on a microbalance with an accuracy of 6 μg (Radwag^®^ MYA 11.4Y, Radom, Poland). The NMR and UV-vis titrations, as well as other experiments using methods nonsensitive to chirality of complexes, were performed only with enantiopure (*R*,*R*)-**cycHC[*n*]** since the same binding for (*S*,*S*)-enantiomers is expected. Titration data were fitted using Bindfit software [46,47]. All the UV-vis, CD, and fluorescence spectra are presented in the range of wavelengths corresponding to absorption or emission of **bis–ZnOEP**, while **cycHC[*n*]**s have no bands in the same range (see Supplementary Materials page S27). The excess of 2000 or more equivalents of **cycHC[*n*]** used in single-addition experiments was necessary to secure sufficient abundance of complex in solution. The amount was rationalized based on the shape of UV-vis titration binding isotherms (seeming saturation in the case of **cycHC[8]**) and mole fractions based on the obtained association constants for **cycHC[6]**. We examined previously obtained crystal structures of **bis–ZnOEP** [51,52,53] and of **cycHC[*n*]** with ZnTPP [36] for their similarity to herein presented complexes. All distances were measured between the centers of corresponding atoms, and the van der Waals radius was then added. 

### 3.2. Spectroscopic Measurements

^1^H-NMR experiments were measured using a QCI CryoProbe and DUL probe on a Bruker AVANCE III 800 MHz (Bruker Corporation, Billerica, MA, USA) spectrometer at a temperature of 298.15 K, except that time-dependent ^1^H-NMR and VT-NMR (253–298 K) were measured using 5 mm ID probe (Inverse Detect probe) on Agilent DD2 500 MHz spectrometer (Agilent Technologies, Inc., Santa Clara, CA, USA). All NMR titrations were performed in either CD_2_Cl_2_ or CH_2_Cl_2_ containing 10% CDCl_3_ to lock. Data was processed with MestreNova (Version 14.1.2) software. The UV-vis absorption spectra were recorded with Varian Cary^®^ 50 UV-vis spectrophotometer (Agilent Technologies, Inc., Santa Clara, CA, USA). The CD spectra were recorded with a Jasco J-1500 circular dichroism spectrophotometer (JASCO International Co., Ltd., Tokyo, Japan). All the fluorescence measurements were recorded by Hitachi F-7000 fluorescence spectrophotometer (Hitachi, Ltd., Tokyo, Japan). All spectroscopic measurements were performed in CH_2_Cl_2_, and concentrations and spectral data are available in further detail in Supplementary Materials.

## 4. Conclusions

In summary, the complex formation of **bis–ZnOEP** with bulky chiral multidentate **cycHC[*n*]** macrocycles was studied using UV–vis, CD, fluorescence, and ^1^H-NMR spectroscopy methods. Although **cycHC[6]** and **cycHC[8]** are chemically analogous and differ only in the number of binding sites, shape, and volume, **bis–ZnOEP** is able to differentiate between the two macrocycles by exhibiting a different behavior related to the self-assembly mechanism. **Bis–ZnOEP** forms a tweezer-like 1:1 complex with **cycHC[6]**, which subsequently transforms into the opened 1:2 *anti* complex upon further addition of **cycHC[6]**. The evaluation of ^1^H-NMR and UV-vis titration data showed a negative cooperativity with the average association constants for both methods: *K_1_* = 3200 M^−1^; *K_2_* = 140 M^−1^. In contrast to **cycHC[6]**, the interaction between **bis–ZnOEP** and **cycHC[8]** exhibited obscure host–guest interaction. This included the intermolecular binding itself and further time-dependent self-association, which prevented the evaluation of the corresponding association constants. This host–guest interaction includes the formation of the 1:1 *syn* complex between **bis–ZnOEP** and **cycHC[8]** followed by slow opening to the *anti* conformation, which is apparently driven by subsequent aggregate generation. Moreover, the specific **cycHC[8]**’s shape and larger number of binding sites leading to a different geometry of the complexes are associated with the presumed aggregate formation, which was not observed in the case of **cycHC[6]** complexes. Finally, this paper confirms that **bis–ZnOEP** is able to recognize **cycHC[*n*]**s and their enantiomers. Chiral **cycHC[6]** induces noticeably intense CD employing exciton coupling, therefore indicating a helical distortion of the whole **bis–ZnOEP** molecule, whilst chiral **cycHC[8]** induces only weak and monosignate CD, with exciton coupling absent apparently due to nearly parallel orientation of the porphyrin cores of **bis–ZnOEP**. In conclusion, this work clearly demonstrated an advanced sensory ability of **bis–ZnOEP** to recognize large macrocyclic systems, such as **cycHC[*n*]**s, and that the complexation-induced aggregation of porphyrins can lead to the formation of new chiral materials.

## Data Availability

Data will be available on reasonable request from the corresponding author.

## Supplementary Materials

The following supporting information can be downloaded online. General information; ^1^H NMR titrations: Figures S1–S7, Tables S1–S3; UV-Vis titrations: Figures S8–S12, Tables S4 and S5; Spectroscopic UV-Vis and fluorescence kinetics: Figures S13–S31; UV-Vis and CD of pure cycHC[n]: The highest intensity UV-Vis maxima for cycHC[n] in CH_3_CN are reported previously in literature [54] and are following: for cycHC[6] λmax = 196 nm and for cycHC[8] λmax = 197 nm. Figure S32; ^1^H NMR time dependent change: Figures S33 and S34; Variable temperature in 1H NMR: Figures S35 and S36; Structural analysis of cycHC and bis–ZnOEP: Figures S37 and S38.

## Sample Availability

Samples of the compounds are available from the corresponding authors.
