# Supplementary material for: Self-Assembly of Chiral Cyclohexanohemicucurbit[n]urils with Bis(Zn Porphyrin): Size, Shape, and Time-Dependent Binding
Source: Molecules. 2022 Jan 29;27(3):937. doi: 10.3390/molecules27030937 (PMC8839860; doi:10.3390/molecules27030937)
Supplement: Supplementary file 1 [file molecules-27-00937-s001.zip › molecules-1484472-supplementary.pdf]

Self-Assembly of Chiral Cyclohexanohemicucurbit[*n*]urils with Bis(Zn Porphyrin): Size, Shape, and Time-Dependent Binding

Marko Šakarašvili†,[1], Lukas Ustrnul†,[1], Elina Suut[1], Jagadeesh Varma Nallaparaju[1], Kamini A. Mishra[1], Nele Konrad[1], Jasper Adamson[2], Victor Borovkov*,[1] and Riina Aav*,[1]

[1] Department of Chemistry and Biotechnology, School of Science, Tallinn University of Technology, Tallinn, Estonia; marko.sakarasvili@taltech.ee (M.Š.), lukas.ustrnul@taltech.ee (L.U.)
[2] Laboratory of Chemical Physics, National Institute of Chemical Physics and Biophysics, Tallinn, Estonia
† - contributed equally
* Correspondence: victor.borovkov@taltech.ee (V.B.), riina.aav@taltech.ee (R.A.)

Contents

1. General information 2
   Specification of compounds 2
   Preparation of samples 2
   NMR measurements 2
   UV-VIS, CD and FS measurements 2
2. ^{1}H NMR titrations 3
3. UV-Vis titrations 11
4. Spectroscopic UV-Vis and fluorescence kinetics 16
6. ^{1}H NMR time dependent change 28
7. Variable temperature in ^{1}H NMR 29
8. Structural analysis of cycHC and bis–ZnOEP 31
   Measurements for bis–ZnOEP 31
   Measurements for cycHC 31

1. General information

Specification of compounds

Unless otherwise stated, all reagents and solvents were purchased from commercial suppliers and used as received. Compounds prepared in our laboratories were **bis-ZnOEP** [1], (*S*,*S*) and (*R*,*R*)-**cycHC[8]**,[2] (*S*,*S*) and (*R*,*R*)-**cycHC[6]** [3]. In all cases according procedures described in literature.

Preparation of samples

All the solutions were prepared using Hamilton® Gastight syringes, those syringes were also used for all the additions during UV-VIS and NMR titrations. For the precise measurement in sample preparation, the mass of solvent and its density were used additionally to volumetric glassware. Samples were weighed on a microbalance with an accuracy of 6 μg (Radwag® MYA 11.4Y, Poland).

NMR measurements

All 1H NMR experiments were measured using a QCI CryoProbe and DUL probe on a Bruker AVANCE III 800 MHz spectrometer at a temperature of 298.15 K, except time-dependent 1H NMR and VT-NMR (253–298 K) were measured using 5 mm ID probe (Inverse Detect probe) on Agilent DD2 500 MHz spectrometer. All NMR titrations were carried out in either CD_2Cl_2 or CH_2Cl_2 containing 10 % $CDCl_3$ to lock. Chemical shifts in ^1H NMR were referenced to $CDCl_3$ residual peak at 7.26 ppm. In all ^1H NMR titrations, concentration of one of the components was kept constant, usually of bis-porphyrin. The number of scans for the ^1H spectra was in the range of 8 to 64 scans and was optimized for the samples to obtain a signal to noise ratio of 250 in the recorded spectra for accurate integration. A relaxation delay of 15 s was used. The acquisition time was set to 2.4 s. The spectra were processed in MestreNova (Version 14.1.2) and zero filled to 128k points to obtain chemical shifts for the titration experiments. For the variable temperature NMR spectra, the sample was allowed to equilibrate at each temperature for 10 minutes before measurements. For Figure 2C and 2D additional data processing was applied by line smoothening and noise cancellation using MestreNova software.

UV-VIS, CD and FS measurements

The UV-VIS absorption spectra were recorded with Varian Cary® 50 UV-Vis spectrophotometer. The CD spectra were recorded with a Jasco J-1500 Circular dichroism spectrophotometer. All spectroscopic measurements were done in CH_2Cl_2.

All the titration experiments were performed in 1 cm screw cap quartz cuvette with PTFE septa, unless specified otherwise. The concentrations of porphyrins were held constant throughout the titration sequence. The stock solution was kept in the vial with PTFE septa and all additions to the cuvette were realized by Hamilton® Gastight syringes to avoid concentration changes caused by the evaporation.

CD measurements were performed in 1 cm screw cap quartz cuvette with PTFE septa or in 0.1 cm cuvette with tight cap. The size of cuvette was chosen according to the used concentration of porphyrin. On all the raw data smoothening procedure was used.

All the fluorescence measurements were recorded by Hitachi F-7000 fluorescence spectrophotometer. The measurements were performed in 1 cm screw cap cuvette with PTFE septa. The data of fluorescence was normalized according to absorption at the excitation wavelength.

2. ^{1}H NMR titrations

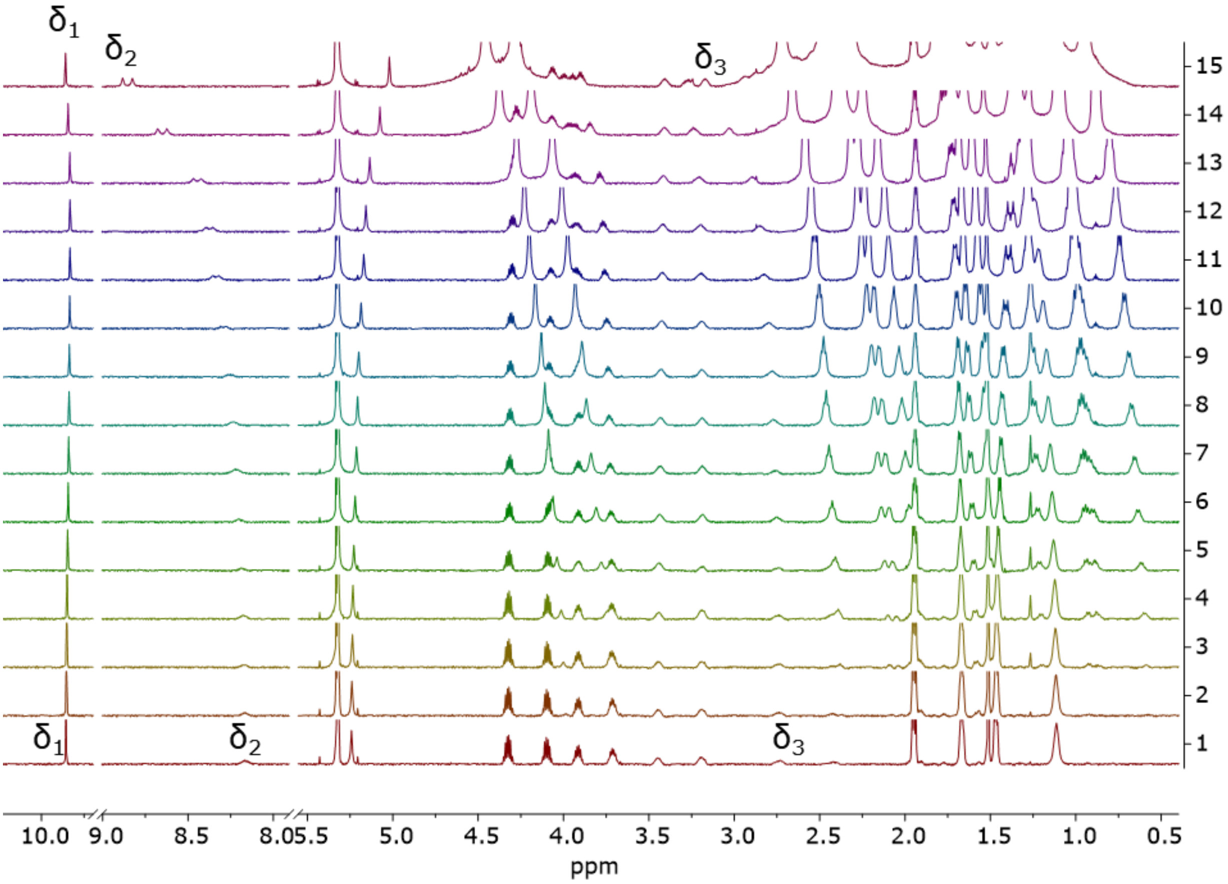

Figure S1 ^{1}H NMR titration of **bis-ZnOEP** (1.05 ·10^{-3} M) with (*R*,*R*)-**cycHC[6]** from 0 to 16 equivalence in CD_2Cl_2 at 298 K. Numbers of spectra (vertical axis) correspond to sample numbers in table S1

Table S1 Chemical shifts (ppm) of **bis-ZnOEP** (1.05·10^{-3} M) obtained from ^{1}H NMR titration of **bis-ZnOEP** with (*R*,*R*)-**cycHC[6]** in CD_2Cl_2 at 298 K (Figure S1).

**Sample no**	**δ_1, ppm**	**δ_2, ppm**	**δ_3, ppm**	**C((*R*,*R*)-cycHC[6]), M**
1	9.857	8.16	2.73	0.00
2	9.854	8.17	2.74	1.06·10^{-4}
3	9.854	8.17	2.74	2.11·10^{-4}
4	9.852	8.18	2.74	3.15·10^{-4}
5	9.848	8.19	2.74	5.25·10^{-4}
6	9.844	8.20	2.75	7.88·10^{-4}
7	9.841	8.22	2.76	1.05·10^{-3}

8	9.839	8.24	2.77	$1.31 \cdot 10^{-3}$
9	9.838	8.26	2.78	$1.58 \cdot 10^{-3}$
10	9.836	8.30	2.80	$2.09 \cdot 10^{-3}$
11	9.834	8.34	2.83	$2.62 \cdot 10^{-3}$
12	9.834	8.38	2.85	$3.16 \cdot 10^{-3}$
13	9.835	8.45	2.90	$4.20 \cdot 10^{-3}$
14	9.845	8.65	3.03	$8.46 \cdot 10^{-3}$
15	9.859	8.85	3.17	$1.68 \cdot 10^{-2}$

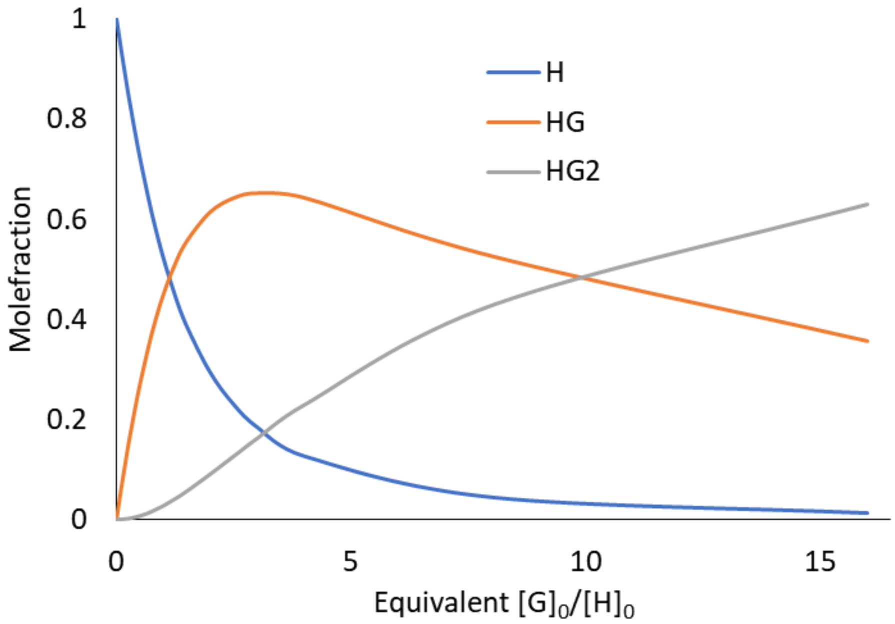

Figure S2 Molefractions of **cycHC[6]** as guest (G) and as **bis-ZnOEP** host (H) obtained from ^{1}H NMR titration (Figure S1, Table S1). $[G]_0/[H]_0$ defines the formal molar ratio of **cycHC[6]** and **bis-ZnOEP** host. Blue line – host, orange line – host guest 1:1 complex, gray line – host guest 1:2 complex. Data fitted using Bindfit [4,5]

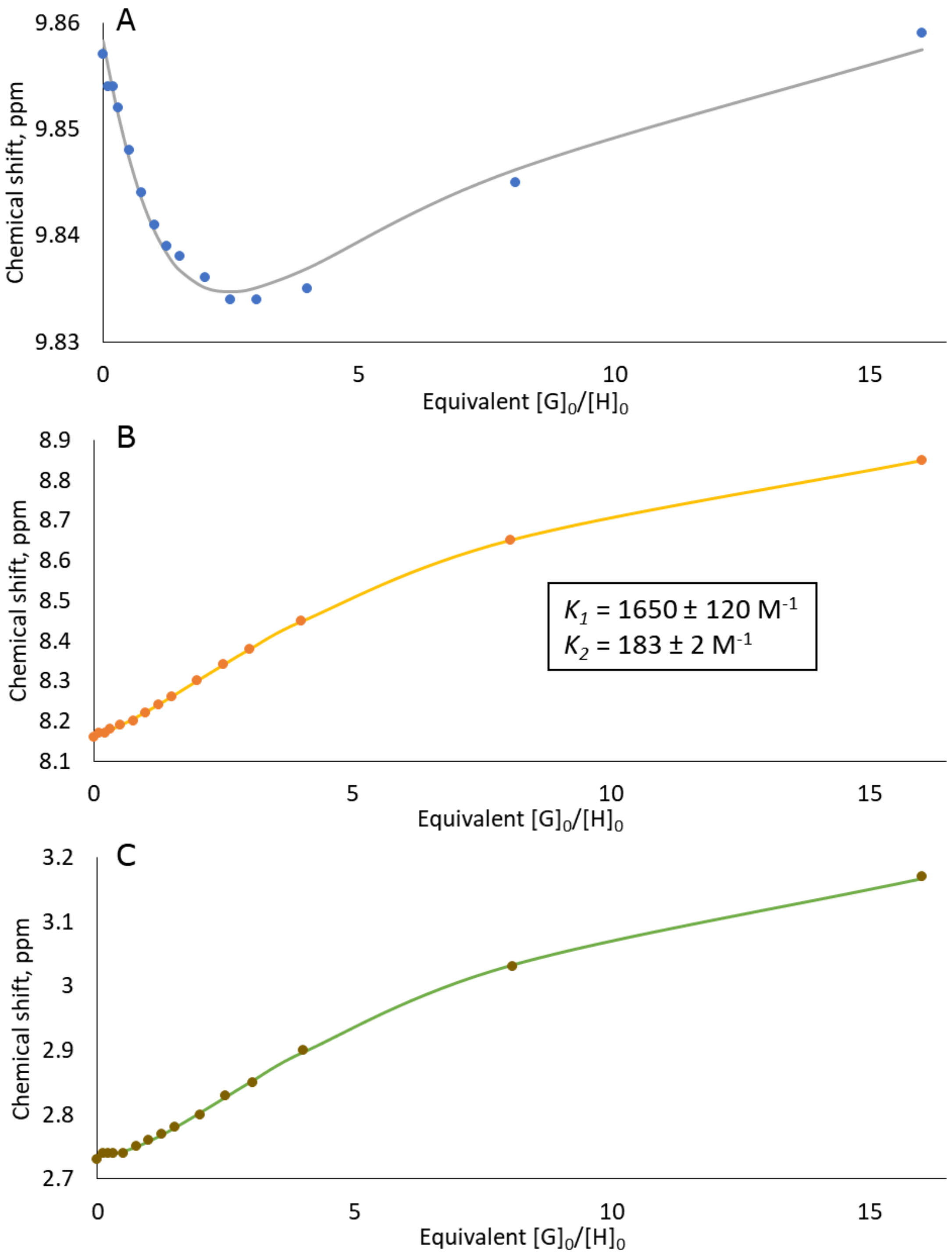

Figure S3 ^{1}H NMR experimental (points) and 1:2 fitted (line) titration curves of **bis-ZnOEP** and **cycHC[6]** in CD_2Cl_2. $[G]_0/[H]_0$ defines the formal molar ratio of **cycHC[6]** guest and **bis-ZnOEP** host. A) Blue points – chemical shifts at 9.857 ppm (δ_1); B) orange points – chemical shifts at 8.16 ppm (δ_2) and C) brown points – brown points at 2.73 ppm (δ_3) (data from table S1). Association constants were obtained by simultaneous fitting of all three signals using Bindfit [4,5]

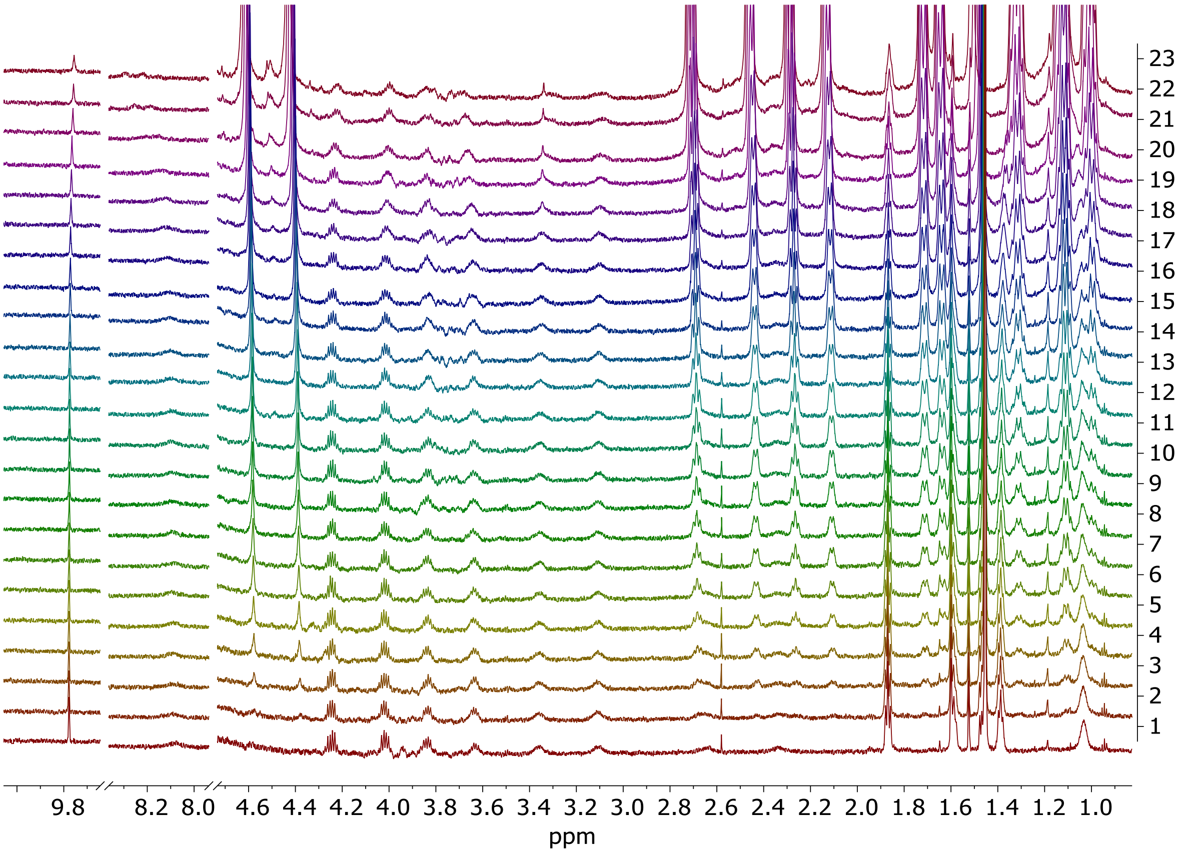

Figure S4 ^{1}H NMR titration of **bis-ZnOEP** (6.6 $\cdot 10^{-4}$ M) with (*R*,*R*)-**cycHC[8]** from 0 to 15 equivalence in CH_2Cl_2 / $CDCl_3$ (V/V 90:10) at 298 K. Numbers of spectra (vertical axis) correspond to sample numbers in table S2

Table S2 Chemical shifts (ppm) of **bis-ZnOEP** ($6.6 \cdot 10^{-4}$ M) obtained from ^{1}H NMR titration of **bis-ZnOEP** with (*R*,*R*)-**cycHC[8]** in CH_2Cl_2 / $CDCl_3$ (V/V 90:10) at 298 K (Figure S3).

**Sample no**	**δ_1, ppm**	**C((*R*,*R*)-cycHC[8]), M**
1	9.778	0.00
2	9.778	$5.86 \cdot 10^{-5}$
3	9.778	$1.17 \cdot 10^{-4}$
4	9.777	$1.77 \cdot 10^{-4}$
5	9.777	$2.38 \cdot 10^{-4}$
6	9.777	$2.97 \cdot 10^{-4}$
7	9.777	$3.56 \cdot 10^{-4}$
8	9.776	$4.15 \cdot 10^{-4}$
9	9.776	$4.74 \cdot 10^{-4}$
10	9.775	$5.34 \cdot 10^{-4}$
11	9.775	$5.95 \cdot 10^{-4}$

12	9.775	$6.59 \cdot 10^{-4}$
13	9.774	$7.90 \cdot 10^{-4}$
14	9.773	$9.89 \cdot 10^{-4}$
15	9.772	$1.19 \cdot 10^{-3}$
16	9.772	$1.39 \cdot 10^{-3}$
17	9.771	$1.64 \cdot 10^{-3}$
18	9.769	$1.98 \cdot 10^{-3}$
19	9.767	$2.64 \cdot 10^{-3}$
20	9.765	$3.31 \cdot 10^{-3}$
21	9.761	$5.28 \cdot 10^{-3}$
22	9.758	$7.26 \cdot 10^{-3}$
23	9.756	$9.88 \cdot 10^{-3}$

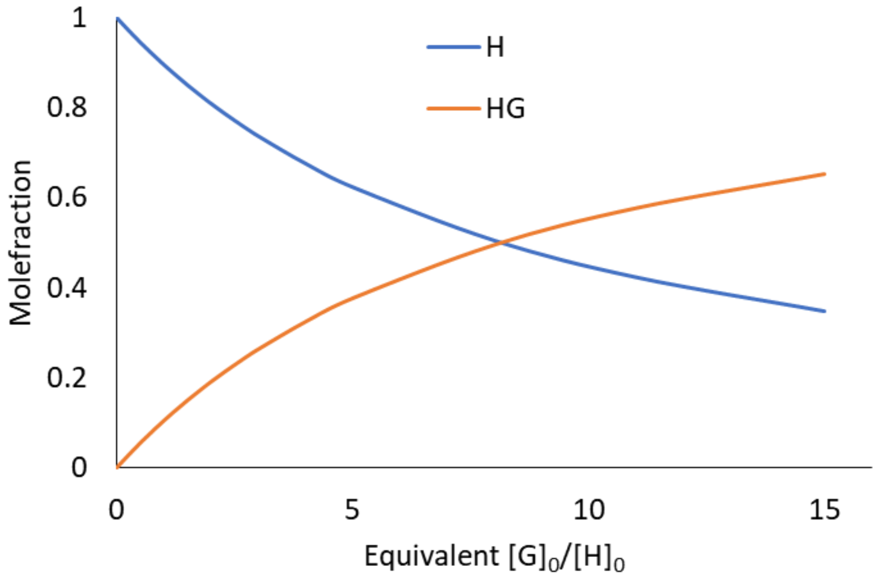

Figure S5 Molefractions of **cycHC[8]** as guest (G) and as **bis-ZnOEP** host (H) obtained from ^{1}H NMR titration (Figure S4, Table S2). $[G]_0/[H]_0$ defines the formal molar ratio of **cycHC[8]** and **bis-ZnOEP** host. Blue line – host, orange line – host guest 1:1 complex. Data fitted using Bindfit [4,5]

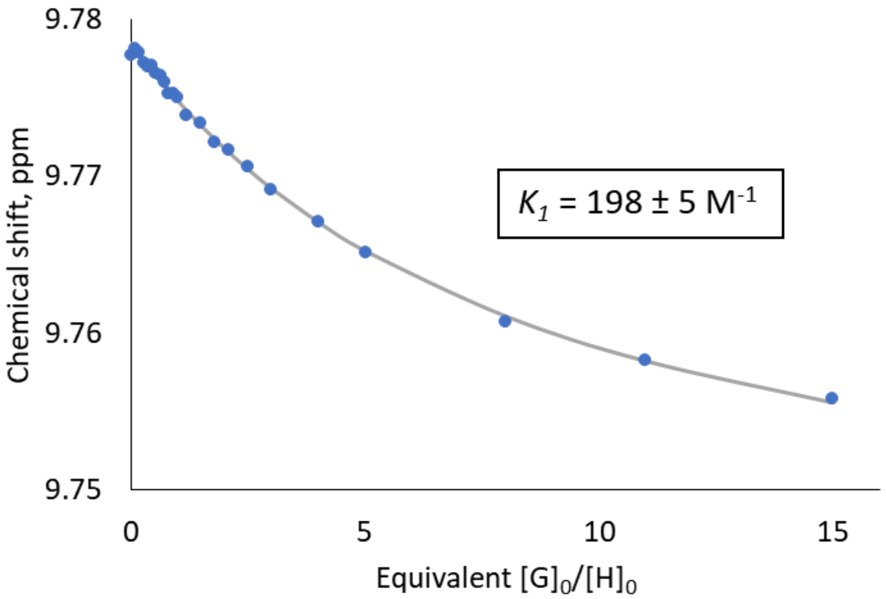

Figure S6 ^{1}H NMR experimental (points) and 1:1 fitted (line) titration curve of **bis-ZnOEP** and **cycHC[8]** in CD_2Cl_2. $[G]_0/[H]_0$ defines the formal molar ratio of **cycHC[8]** guest and **bis-ZnOEP** host. A) Blue points – chemical shifts at 9.778 ppm (δ_1). Association constants were obtained by using Bindfit [4,5]

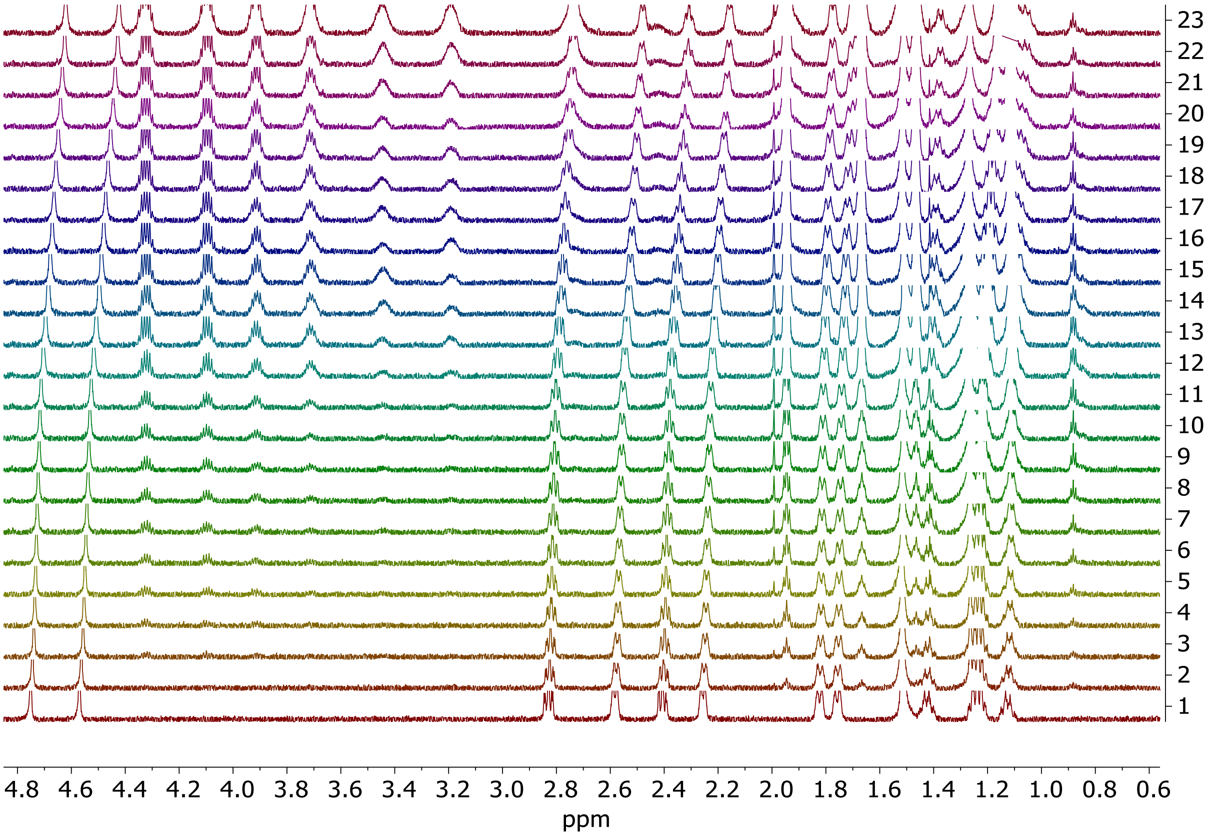

Figure S7 ^{1}H NMR titration of (*R*,*R*)-**cycHC[8]** ($1.93 \cdot 10^{-4}$ M) with **bis-ZnOEP** from 0 to 4.25 equivalence in CD_2Cl_2 at 298 K. Numbers of spectra (vertical axis) correspond to sample numbers in table S3

Table S3 Chemical shifts (ppm) of (*R*,*R*)-**cycHC[8]** (1.93·10^{-4} M) obtained from ^{1}H NMR titration of (*R*,*R*)-**cycHC[8]** with **bis-ZnOEP** in CD_2Cl_2 at 298 K (Figure S5)

Sample no	δ_1, ppm	δ_2, ppm	δ_3, ppm	δ_4, ppm	C(bis-ZnOEP), M
1	4.750	4.570	2.830	2.256	0.00
2	4.744	4.562	2.825	2.252	1.98·10^{-5}
3	4.739	4.557	2.822	2.248	7.82·10^{-5}
4	4.735	4.553	2.819	2.245	9.77·10^{-5}
5	4.732	4.549	2.817	2.243	1.17·10^{-4}
6	4.729	4.546	2.814	2.240	1.37·10^{-4}
7	4.726	4.542	2.812	2.238	1.56·10^{-4}
8	4.723	4.538	2.810	2.235	1.76·10^{-4}
9	4.719	4.535	2.807	2.233	1.95·10^{-4}
10	4.716	4.531	2.805	2.230	2.15·10^{-4}
11	4.712	4.526	2.802	2.227	2.44·10^{-4}
12	4.704	4.517	2.796	2.221	2.92·10^{-4}
13	4.695	4.507	2.789	2.214	3.42·10^{-4}
14	4.686	4.497	2.783	2.207	3.91·10^{-4}
15	4.678	4.488	2.777	2.201	4.39·10^{-4}
16	4.671	4.480	2.772	2.196	4.88·10^{-4}
17	4.664	4.472	2.768	2.187	5.37·10^{-4}
18	4.656	4.464	2.762	2.185	5.86·10^{-4}
19	4.648	4.454	2.756	2.178	6.35·10^{-4}
20	4.640	4.445	2.750	2.172	6.85·10^{-4}
21	4.633	4.437	2.742	2.163	7.33·10^{-4}

22	4.625	4.428	2.738	2.159	$7.82 \cdot 10^{-4}$
23	4.621	4.423	2.737	2.157	$8.23 \cdot 10^{-4}$

3. UV-Vis titrations

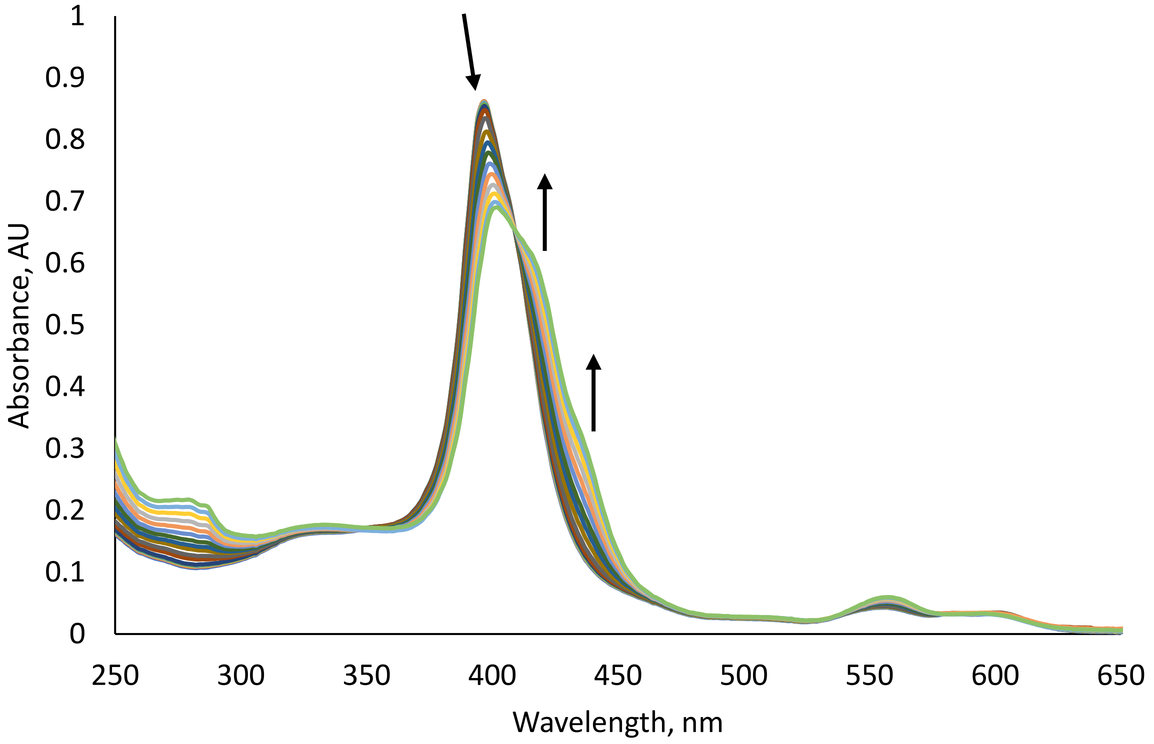

Figure S8 Changes of **bis-ZnOEP** solution ($3.2\cdot10^{-6}$ M, CH_2Cl_2, 296 K) absorption spectrum caused by portion wise addition of (*R*,*R*)-**cycHC[6]** from 0 to 2002 equivalence

Table S4 Absorptions (AU) of **bis-ZnOEP** ($3.2\cdot10^{-6}$ M) obtained from UV-Vis titration of **bis-ZnOEP** with (*R*,*R*)-**cycHC[6]** in CH_2Cl_2 (Figure S6).

**Sample no**	**397.0 nm, AU**	**418.0 nm, AU**	**436.0 nm, AU**	**C((*R*,*R*)-cycHC[6]), M**
1	0.862	0.411	0.127	0.00
2	0.862	0.415	0.129	$9.55\cdot10^{-6}$
3	0.861	0.416	0.129	$1.91\cdot10^{-5}$
4	0.860	0.417	0.129	$2.86\cdot10^{-5}$
5	0.860	0.419	0.130	$4.76\cdot10^{-5}$
6	0.857	0.422	0.132	$7.97\cdot10^{-5}$
7	0.854	0.426	0.134	$1.28\cdot10^{-4}$
8	0.848	0.432	0.138	$2.43\cdot10^{-4}$

9	0.835	0.445	0.148	$4.88 \cdot 10^{-4}$
10	0.811	0.465	0.166	$9.75 \cdot 10^{-4}$
11	0.789	0.484	0.183	$1.46 \cdot 10^{-3}$
12	0.769	0.499	0.200	$1.94 \cdot 10^{-3}$
13	0.744	0.519	0.221	$2.58 \cdot 10^{-3}$
14	0.721	0.536	0.242	$3.23 \cdot 10^{-3}$
15	0.695	0.554	0.263	$4.05 \cdot 10^{-3}$
16	0.674	0.569	0.283	$4.85 \cdot 10^{-3}$
17	0.654	0.582	0.302	$5.65 \cdot 10^{-3}$
18	0.640	0.594	0.317	$6.45 \cdot 10^{-3}$

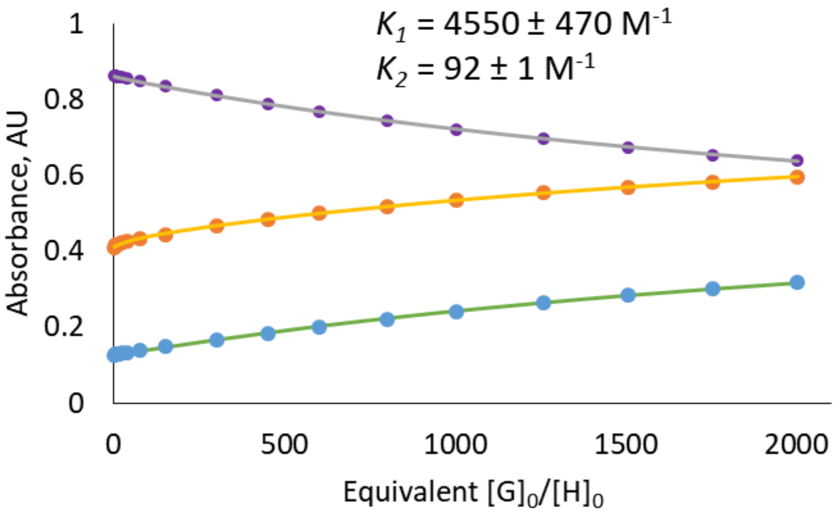

Figure S9 UV-Vis experimental (points) and 1:2 fitted (line) titration curves of **bis-ZnOEP** and **cycHC[6]** in CH_2Cl_2. $[G]_0/[H]_0$ defines the ratio of **cycHC[6]** guest and **bis-ZnOEP** host. Purple points – absorbance at 397.0 nm, orange points – absorbance at 418.0 nm and blue points – absorbance at 436.0 nm (data from table S4). Data fitted using Bindfit **[4,5]**

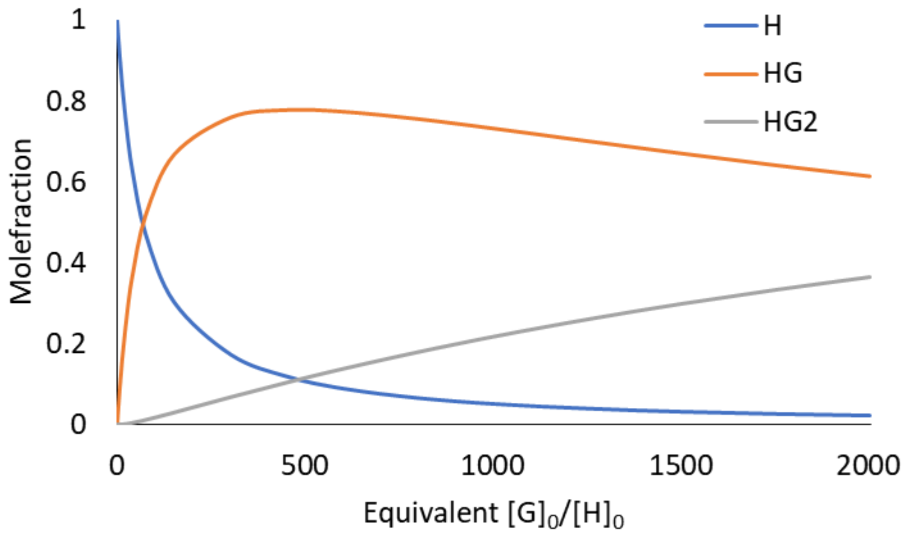

Figure S10 Molefractions of **cycHC[6]** guest and **bis-ZnOEP** host UV-Vis titration. $[G]_0/[H]_0$ defines the ratio of **cycHC[6]** guest and **bis-ZnOEP** host. Blue line – host, orange line – host guest 1:1 complex, gray line – host guest 1:2 complex. Data fitted using Bindfit **[4,5]**

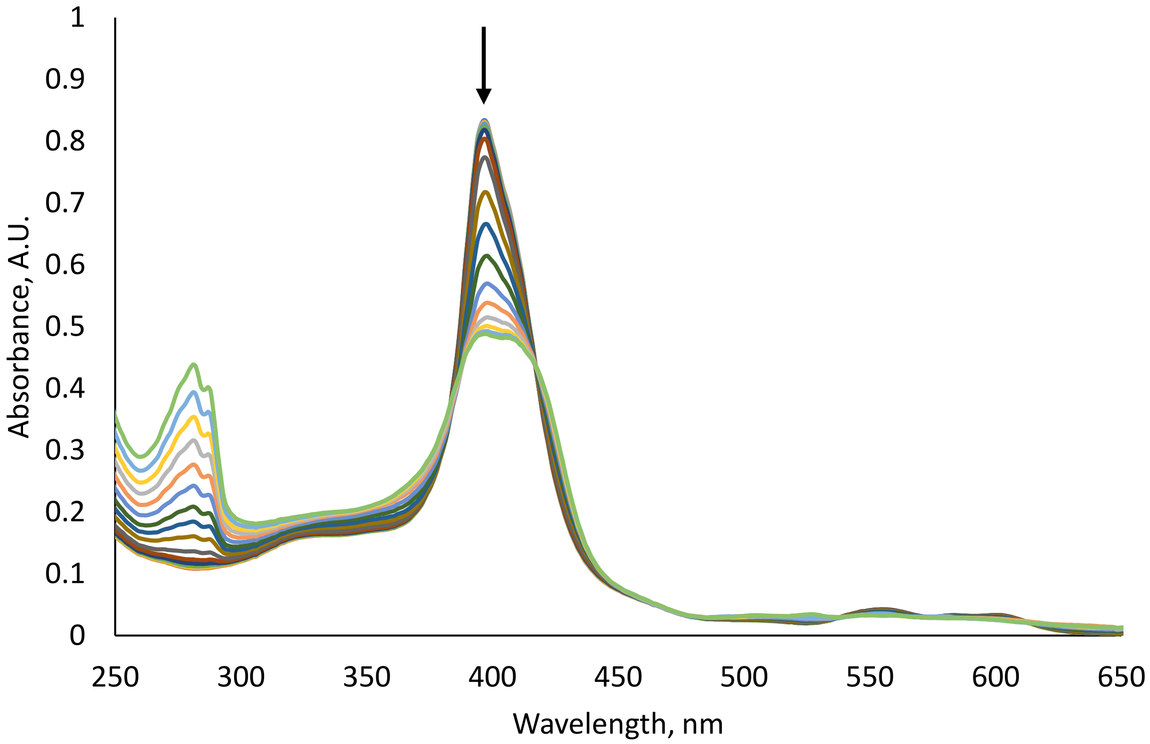

Figure S11 Changes of **bis-ZnOEP** solution ($3.2 \cdot 10^{-6}$ M, CH_2Cl_2, 296 K) absorption spectrum caused by portion wise addition of (*R*,*R*)-**cycHC[8]** from 0 to 2040 equivalence.

Table S5 Absorptions (AU) of **bis-ZnOEP** ($3.2 \cdot 10^{-6}$ M) derivied from UV-Vis titration of **bis-ZnOEP** with (*R*,*R*)-**cycHC[8]** in CH_2Cl_2 (Figure S8)

Sample no	397.0 nm, AU	C((*R*,*R*)-cycHC[8]), M

1	0.834	0.00
2	0.832	$9.68\cdot10^{-6}$
3	0.831	$1.94\cdot10^{-5}$
4	0.830	$2.90\cdot10^{-5}$
5	0.828	$4.83\cdot10^{-5}$
6	0.824	$8.01\cdot10^{-5}$
7	0.818	$1.28\cdot10^{-4}$
8	0.804	$2.37\cdot10^{-4}$
9	0.774	$4.79\cdot10^{-4}$
10	0.718	$9.53\cdot10^{-4}$
11	0.666	$1.43\cdot10^{-3}$
12	0.613	$1.90\cdot10^{-3}$
13	0.568	$2.53\cdot10^{-3}$
14	0.537	$3.18\cdot10^{-3}$
15	0.514	$3.97\cdot10^{-3}$
16	0.500	$4.76\cdot10^{-3}$
17	0.492	$5.56\cdot10^{-3}$
18	0.488	$6.49E\cdot10^{-3}$

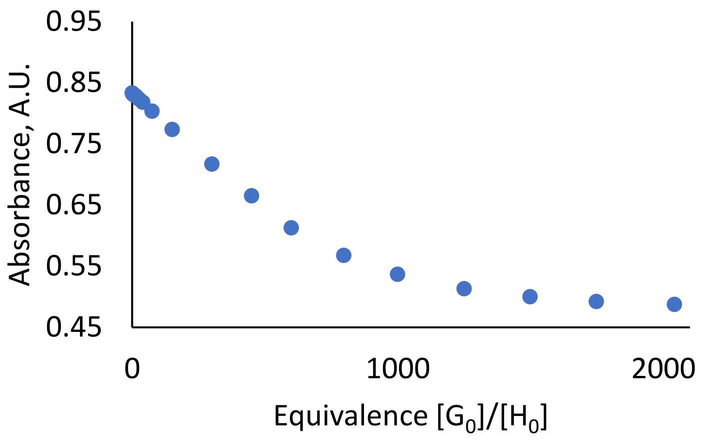

Figure S12 UV-vis experimental points at 397.0 nm from titration of **bis-ZnOEP** and **cycHC[8]** in CH_2Cl_2. $[G]_0/[H]_0$ defines the ratio of **cycHC[8]** guest and **bis-ZnOEP** host (data from table S5)

4. Spectroscopic UV-Vis and fluorescence kinetics

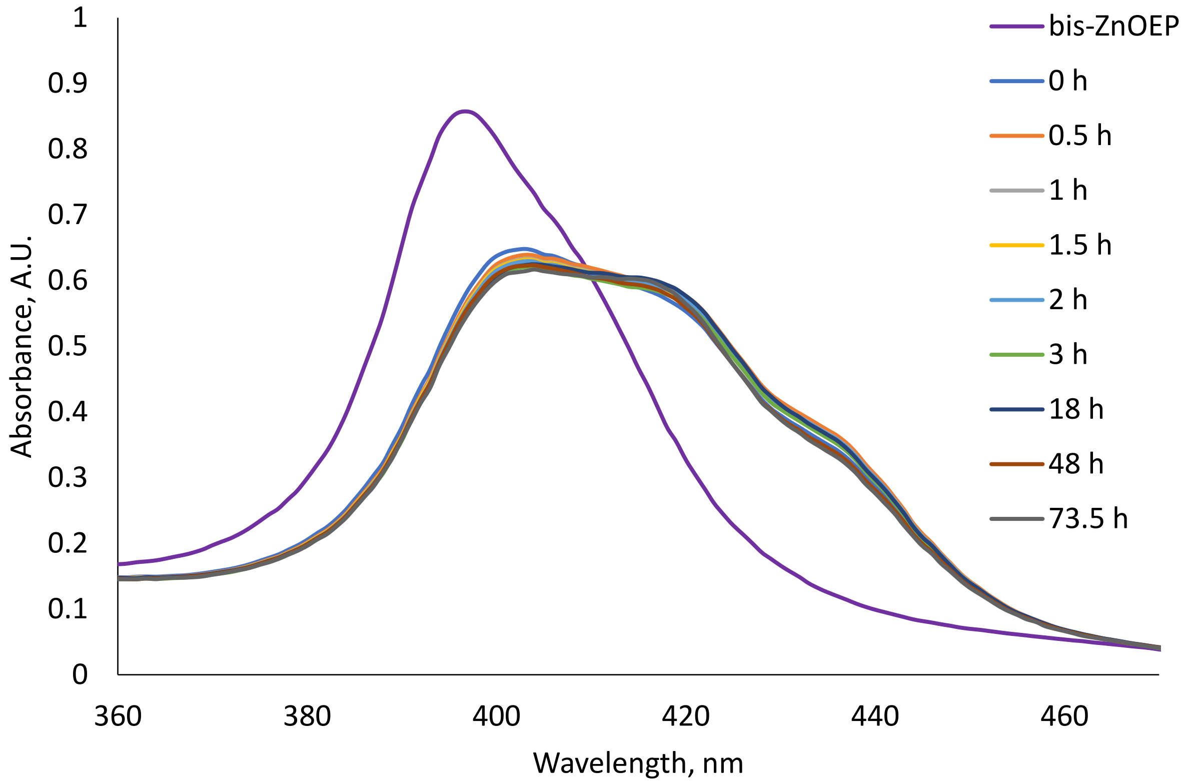

Figure S13 Absorption spectrum of $3.4 \cdot 10^{-5}$ M **bis-ZnOEP** solution in CH_2Cl_2 (purple line) inside a 1 mm cuvette, spectrum after single addition of 247 equivalence of (*R*,*R*)-**cycHC[6]** (0 h line) and its change in time

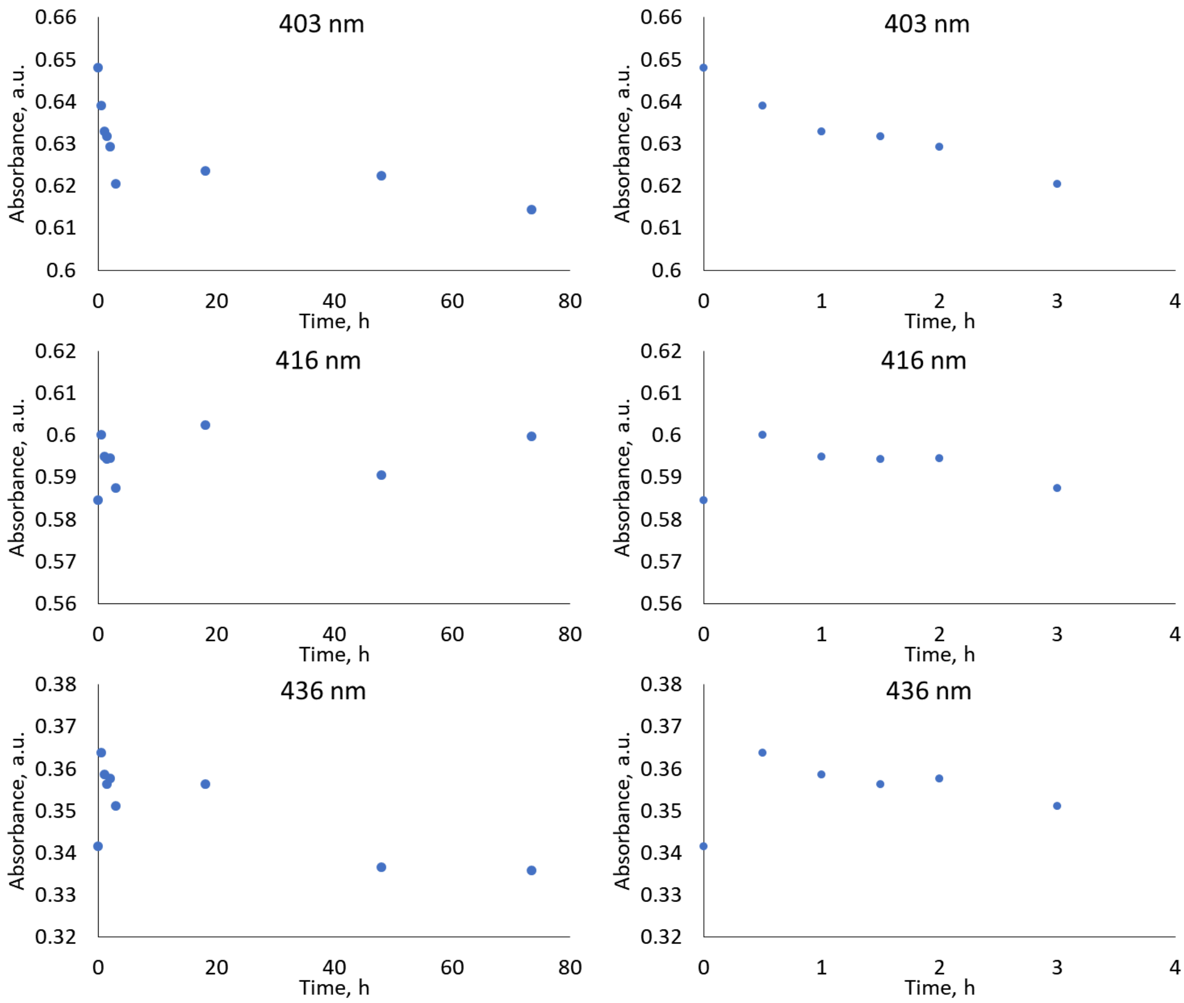

Figure S14 On the left side; change of absorption for selected wavelengths from Figure S9 in time (up to 80 hours). On the right side; enlarged region for period up to 4 hours.

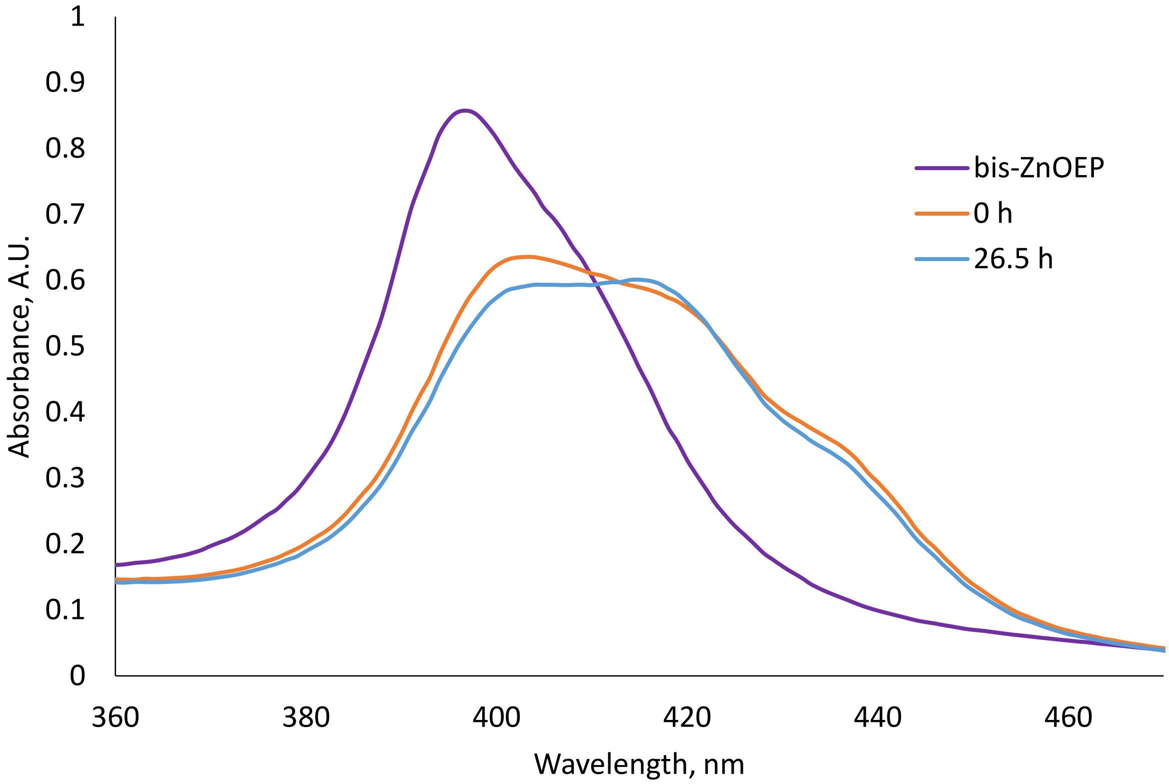

Figure S15 Absorption spectrum of 3.4·10^{-5} M **bis-ZnOEP** solution in CH_2Cl_2 (purple line) inside a 1 mm cuvette, spectrum after single addition of 246 equivalence of (*S*,*S*)-**cycHC[6]** (0 h line) and its change in time

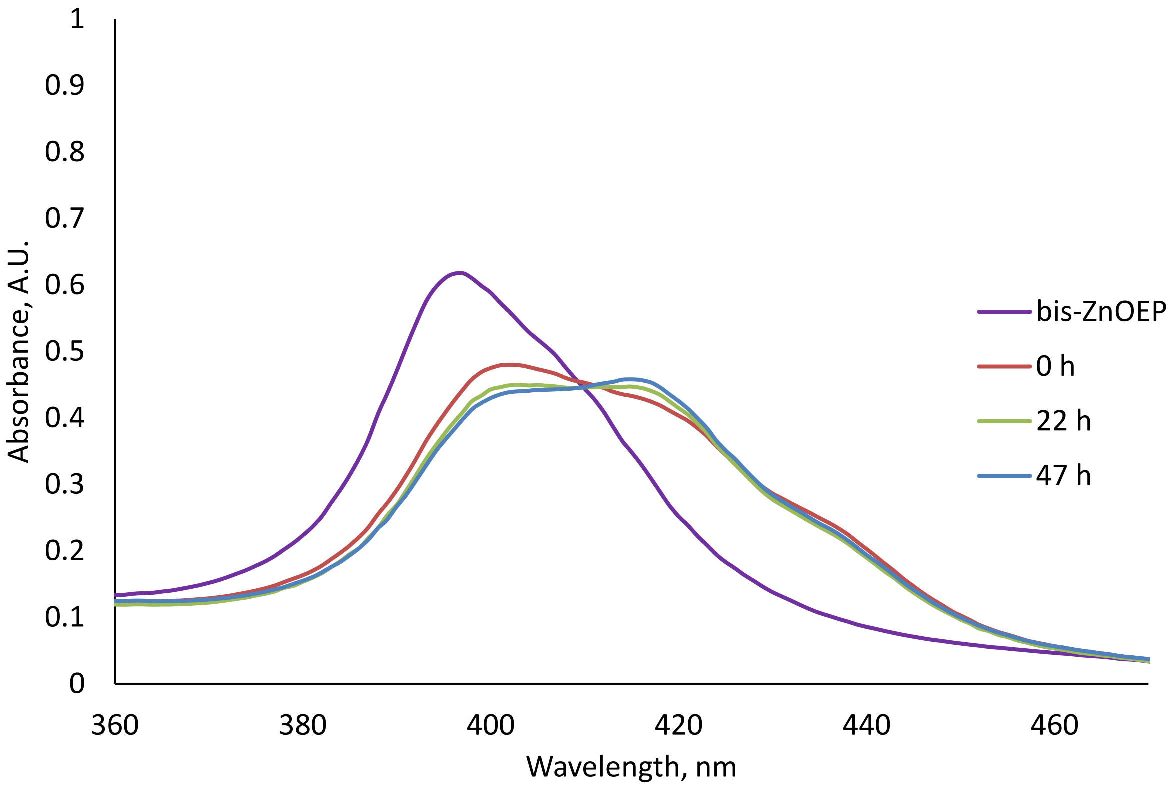

Figure S16 Absorption spectrum of 2.2·10^{-6} M **bis-ZnOEP** solution in CH_2Cl_2 (purple line) inside a 1 cm cuvette, spectrum after single addition of 3040 equivalence of (*R*,*R*)-**cycHC[6]** (0 h line) and its change in time

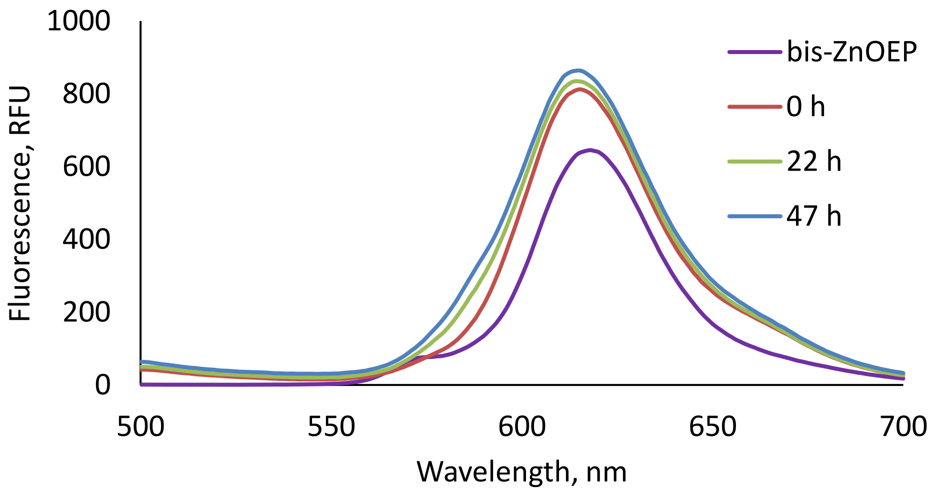

Figure S17 Fluorescence emission spectrum of 2.2·10^{-6} M **bis-ZnOEP** solution in CH_2Cl_2 (purple line), excited at 396 nm inside a 1 cm cuvette, spectrum after single addition of 3040 equivalence of (*R*,*R*)-**cycHC[6]** (0 h line) and its change in time

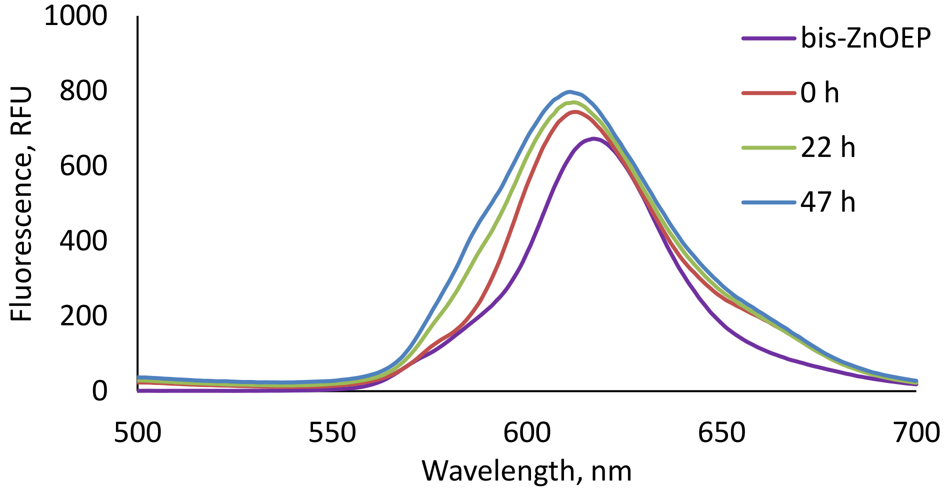

Figure S18 Fluorescence emission spectrum of 2.2·10^{-6} M **bis-ZnOEP** solution in CH_2Cl_2 (purple line), excited at 404 nm inside a 1 cm cuvette, spectrum after single addition of 3040 equivalence of (*R*,*R*)-**cycHC[6]** (0 h line) and its change in time

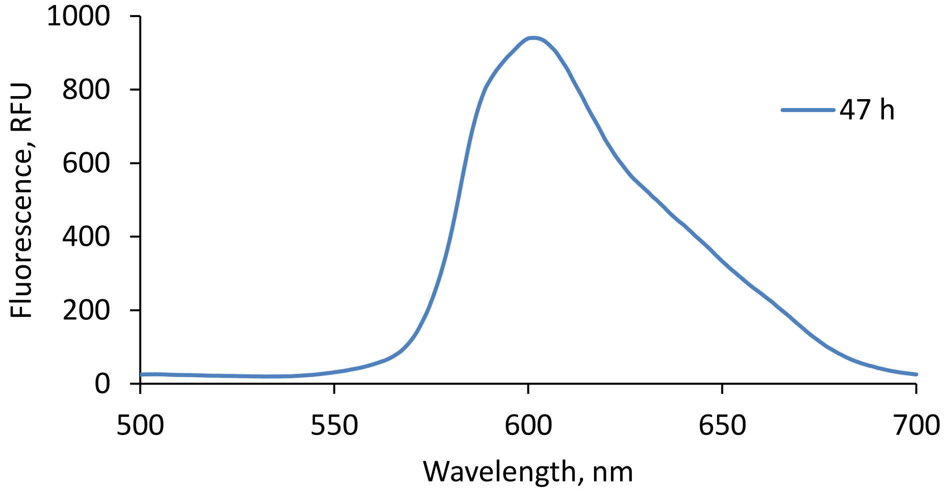

Figure S19 Fluorescence emission spectrum of 2.2·10^{-6} M **bis-ZnOEP** and 3040 equivalence of (*R*,*R*)-**cycHC[6]** solution in CH_2Cl_2, excited at 413 nm inside a 1 cm cuvette at 47 hours after addition

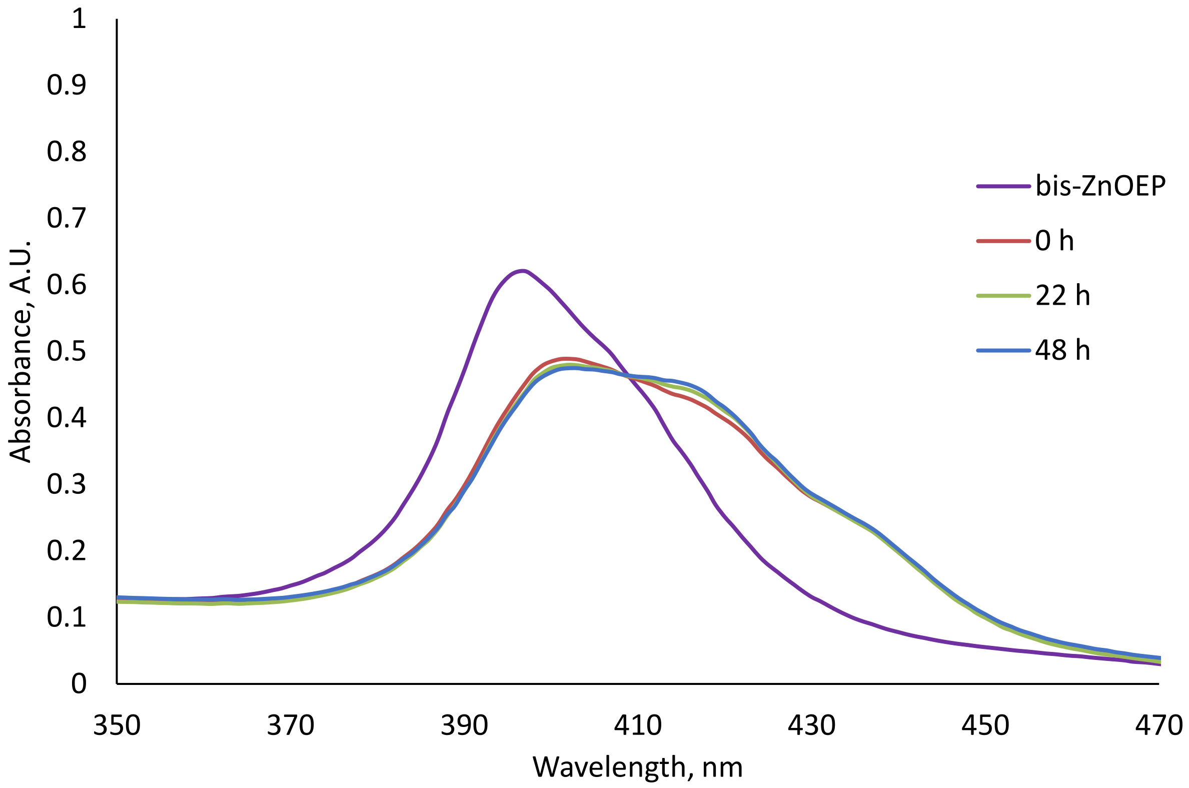

Figure S20 Absorption spectrum of $2.2{\cdot}10^{-6}$ M **bis-ZnOEP** solution in CH_2Cl_2 (purple line) inside a 1 cm cuvette, spectrum after single addition of 2793 equivalence of (*S*,*S*)-**cycHC[6]** (0 h line) and its change in time

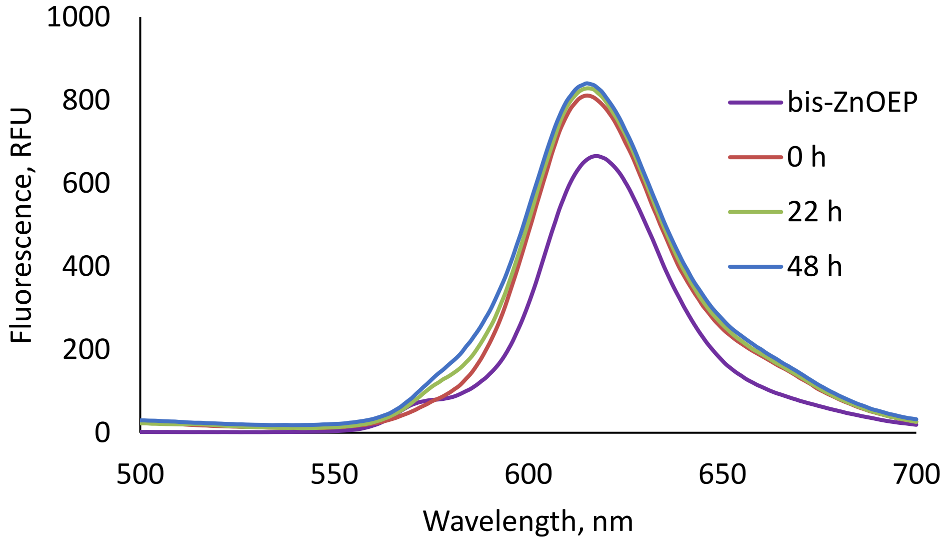

Figure S21 Fluorescence emission spectrum of $2.2{\cdot}10^{-6}$ M **bis-ZnOEP** solution in CH_2Cl_2 (purple line), excited at 396 nm inside a 1 cm cuvette, spectrum after single addition of 2793 equivalence of (*S*,*S*)-**cycHC[6]** (0 h line) and its change in time

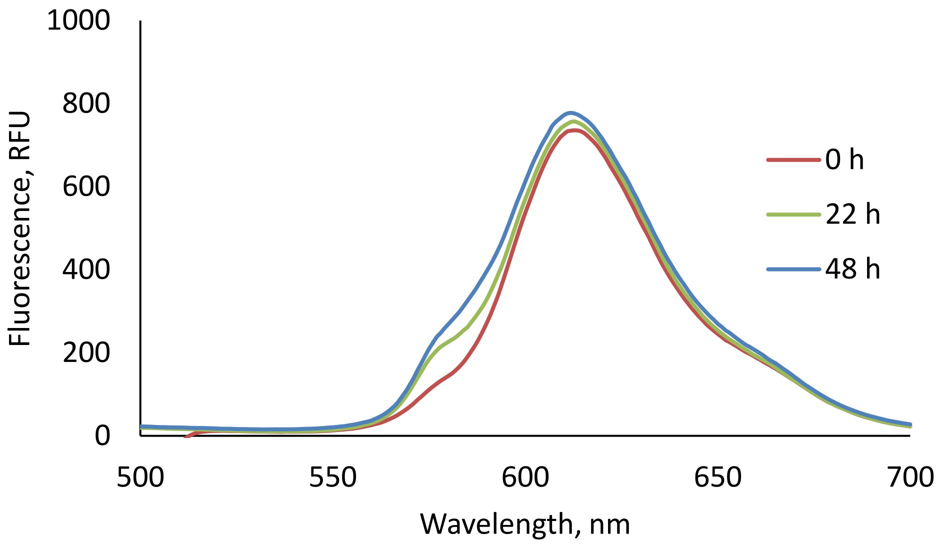

Figure S22 Fluorescence emission spectrum of 2.2·10^{-6} M **bis-ZnOEP** and 2793 equivalence of (*S*,*S*)-**cycHC[6]** solution in CH_2Cl_2, excited at 404 nm inside a 1 cm cuvette, and its change in time

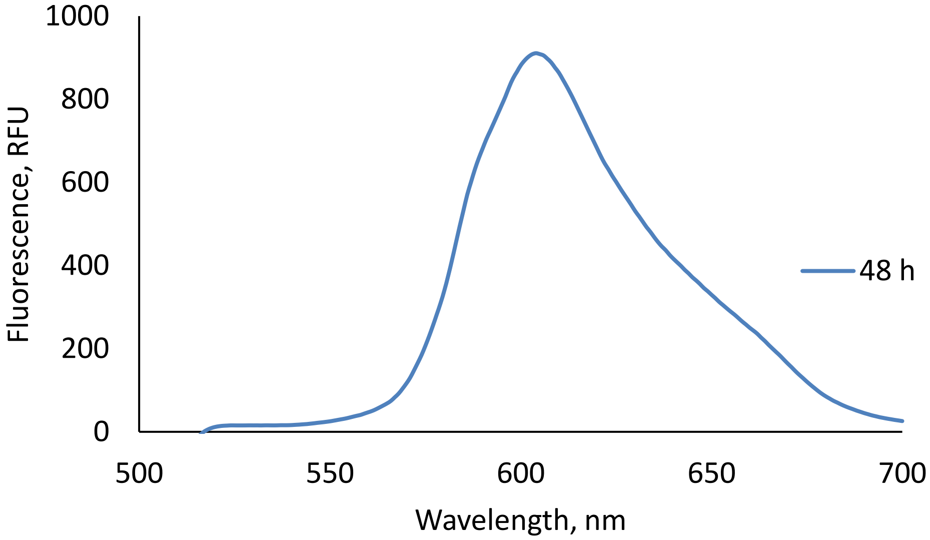

Figure S23 Fluorescence emission spectrum of 2.2·10^{-6} M **bis-ZnOEP** and 2793 equivalence of (*S*,*S*)-**cycHC[6]** solution in CH_2Cl_2, excited at 413 nm inside a 1 cm cuvette at 48 hours after addition

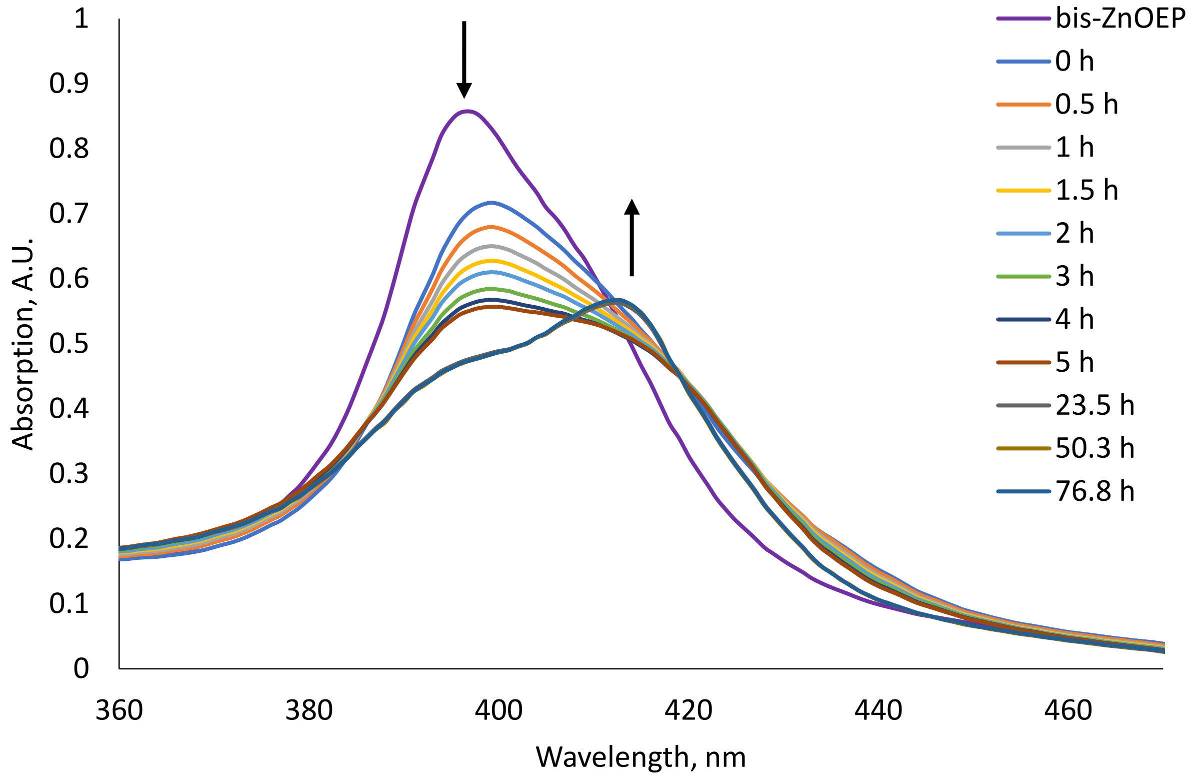

Figure S24 Absorption spectrum of 3.4·10^{-5} M **bis-ZnOEP** solution in CH_2Cl_2 (purple line) inside a 1 mm cuvette, spectrum after single addition of 243 equivalence of (*R*,*R*)-**cycHC[8]** (0 h line) and its change in time

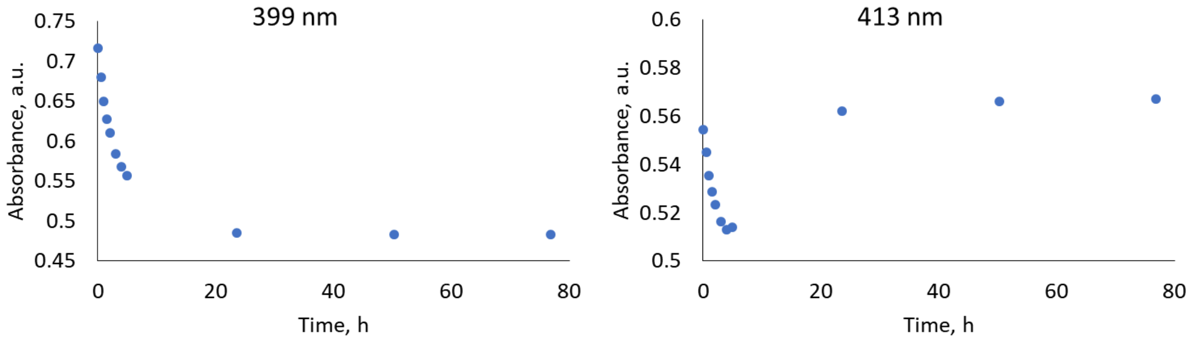

Figure S25 Change of absorption for selected wavelengths from Figure S20 in time (up to 80 hours).

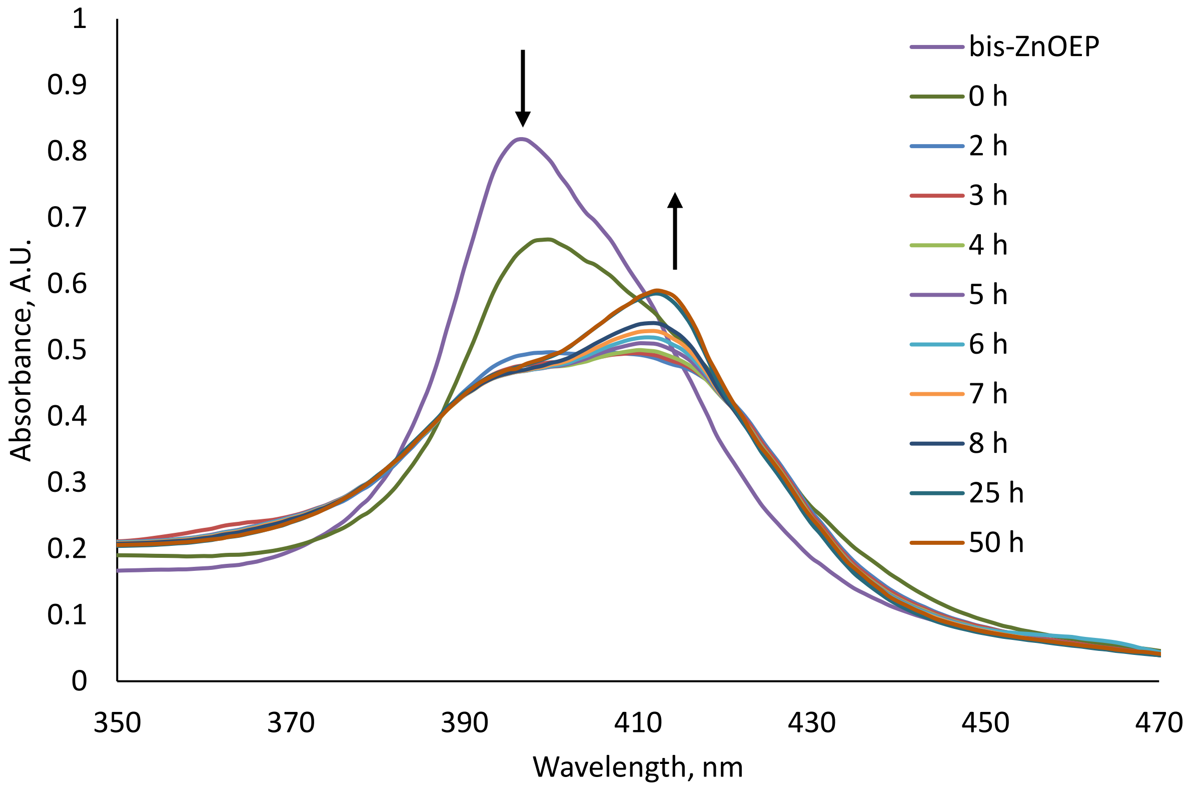

Figure S26 Absorption spectrum of 3.0·10^{-6} M **bis-ZnOEP** solution in CH_2Cl_2 (purple line) inside a 1 cm cuvette, spectrum after single addition of 2166 equivalence of (*R*,*R*)-**cycHC[8]** (0 h line) and its change in time

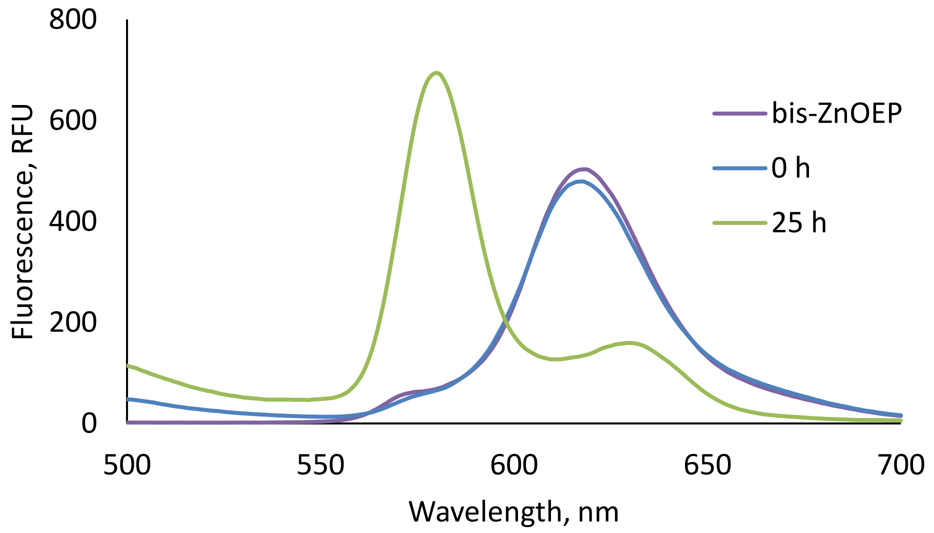

Figure S27 Fluorescence emission spectrum of 3.0·10^{-6} M **bis-ZnOEP** solution in CH_2Cl_2 (purple line), excited at 396 nm inside a 1 cm cuvette, spectrum after single addition of 2166 equivalence of (*R*,*R*)-**cycHC[8]** (0 h line) and its change in time

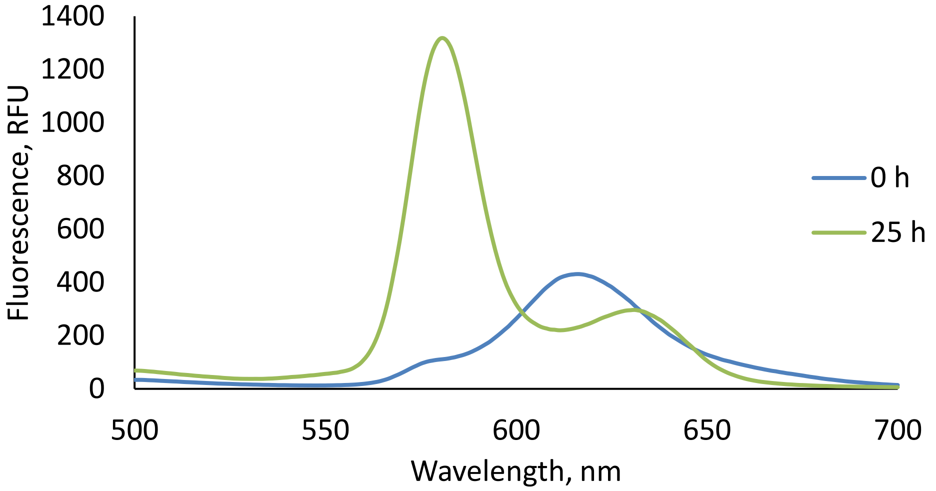

Figure S28 Fluorescence emission spectrum of 3.0·10^{-6} M **bis-ZnOEP** and 2166 equivalence of (*R*,*R*)-**cycHC[8]** solution in CH_2Cl_2, excited at 413 nm inside a 1 cm cuvette right after addition and at 25 hours after addition

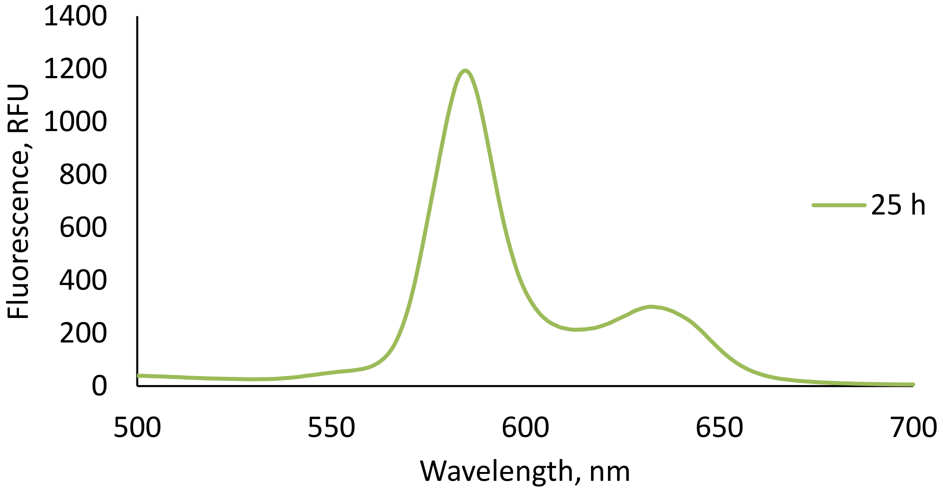

Figure S29 Fluorescence emission spectrum of 3.0·10^{-6} M **bis-ZnOEP** and 2166 equivalence of (*R*,*R*)-**cycHC[8]** solution in CH_2Cl_2, excited at 413 nm inside a 1 cm cuvette at 25 hours after addition

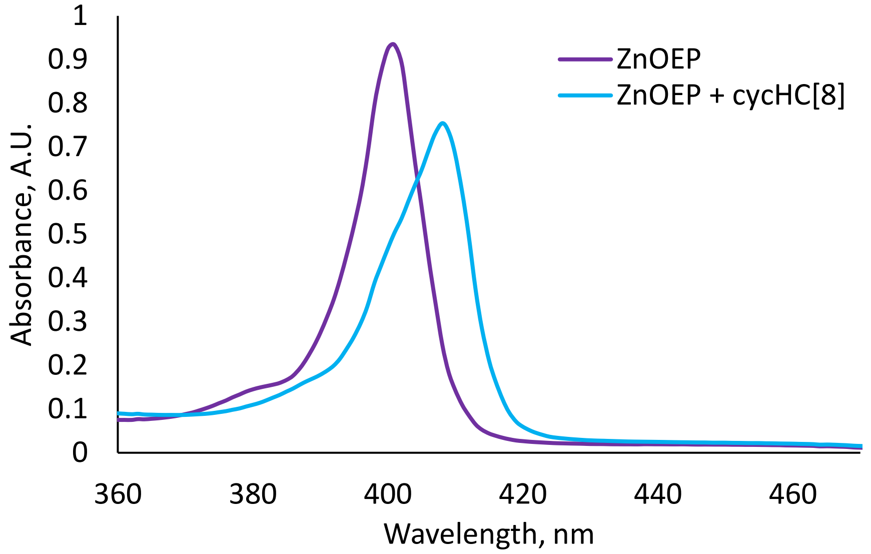

Figure S30 Absorption spectrum of 2.4·10^{-6} M **ZnOEP** solution in CH_2Cl_2 (purple line) inside a 1 cm cuvette, spectrum after single addition of 2231 equivalence of (*S*,*S*)-**cycHC[8]** (blue line)

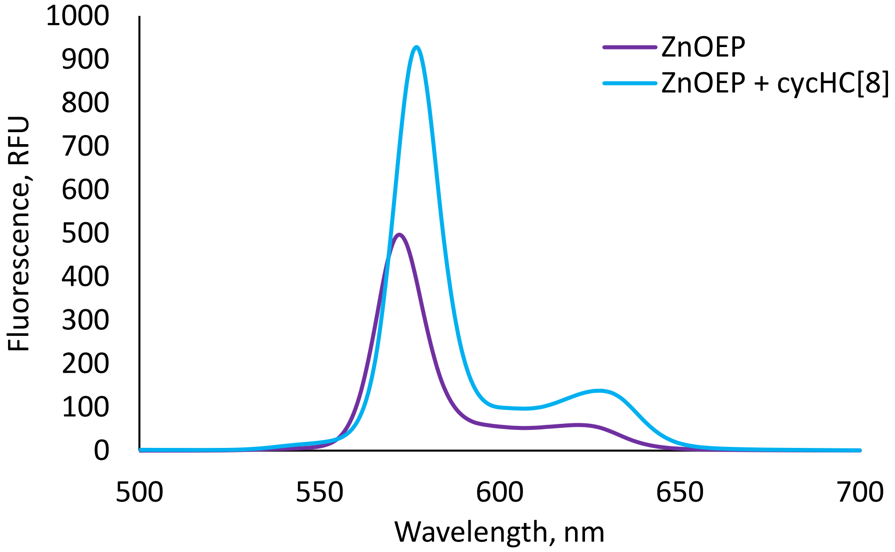

Figure S31 Fluorescence emission spectrum of 2.4·10^{-6} M **ZnOEP** solution in CH_2Cl_2 (purple line), excited at 408 nm inside a 1 cm cuvette and spectrum after single addition of 2167 equivalence of (*S*,*S*)-**cycHC[8]** (blue line)

5. UV-Vis and CD of pure cycHC[*n*]

The highest intensity UV-Vis maxima for **cycHC[*n*]** in CH_3CN are reported previously in literature [6] and are following: for **cycHC[6]** λ_{max}=196 nm and for **cycHC[8]** λ_{max}=197 nm.

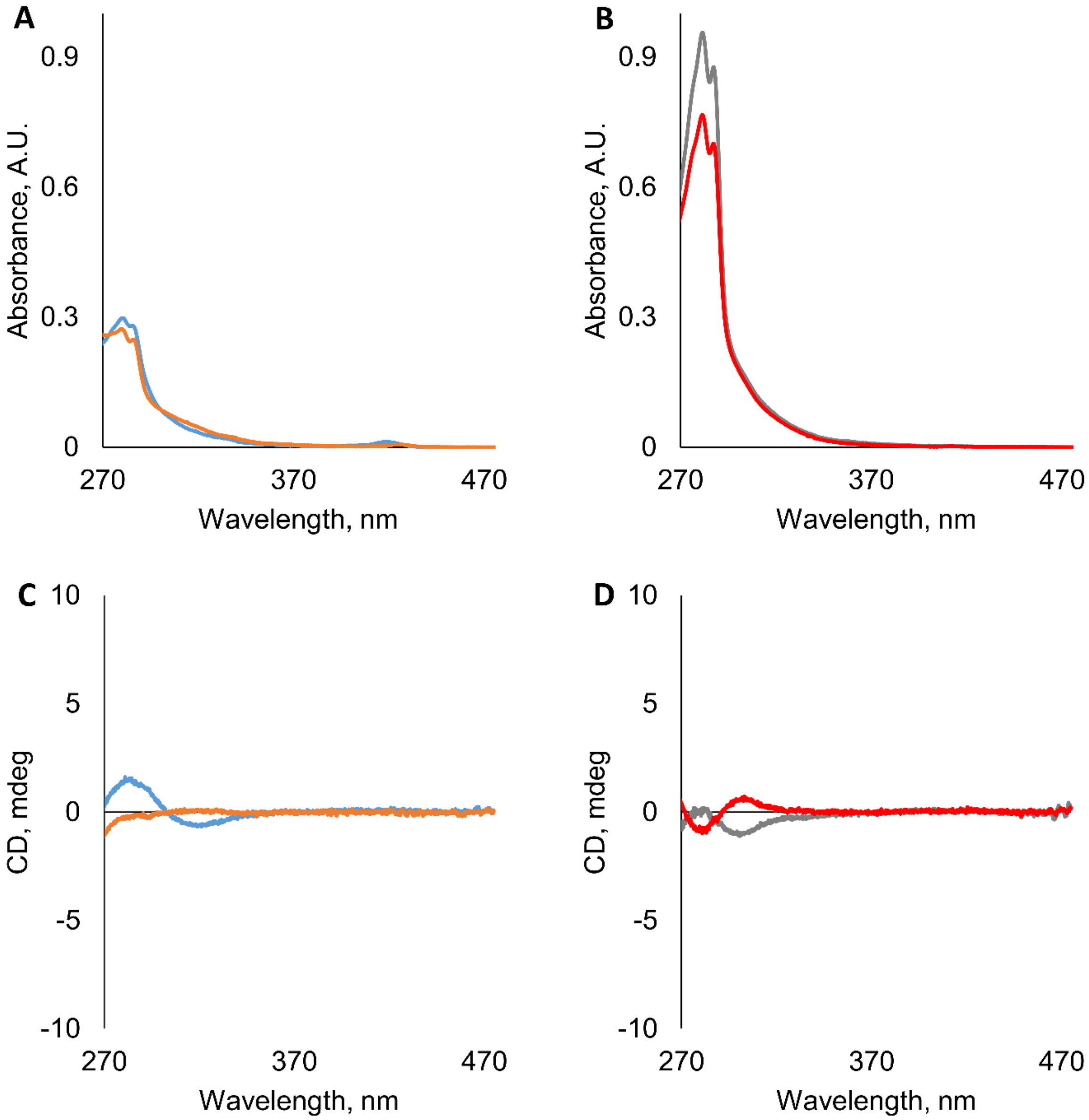

Figure S32 UV-Vis and CD spectra of **cycHC[*n*]** in CH_2Cl_2 at 20 °C. A) UV-Vis spectra of (*R*,*R*)-**cycHC[6]** ($1.5 \cdot 10^{-2}$ M, blue line) and (*S*,*S*)-**cycHC[6]** ($4.0 \cdot 10^{-2}$ M, sample contains CH_3OH, orange line); B) CD spectra of (*R*,*R*)-**cycHC[6]** ($1.5 \cdot 10^{-2}$ M, blue line) and (*S*,*S*)-**cycHC[6]** ($4.0 \cdot 10^{-2}$ M, sample contains CH_3OH, orange line); C) UV-Vis spectra of (*R*,*R*)-**cycHC[8]** ($1.5 \cdot 10^{-2}$ M, gray line) and (*S*,*S*)-**cycHC[8]** ($1.7 \cdot 10^{-2}$ M, sample contains CH_3OH, red line); D) CD spectra of (*R*,*R*)-**cycHC[8]** ($1.5 \cdot 10^{-2}$ M, gray line) and (*S*,*S*)-**cycHC[8]** ($1.7 \cdot 10^{-2}$ M, sample contains CH_3OH, red line).

6. ^{1}H NMR time dependent change

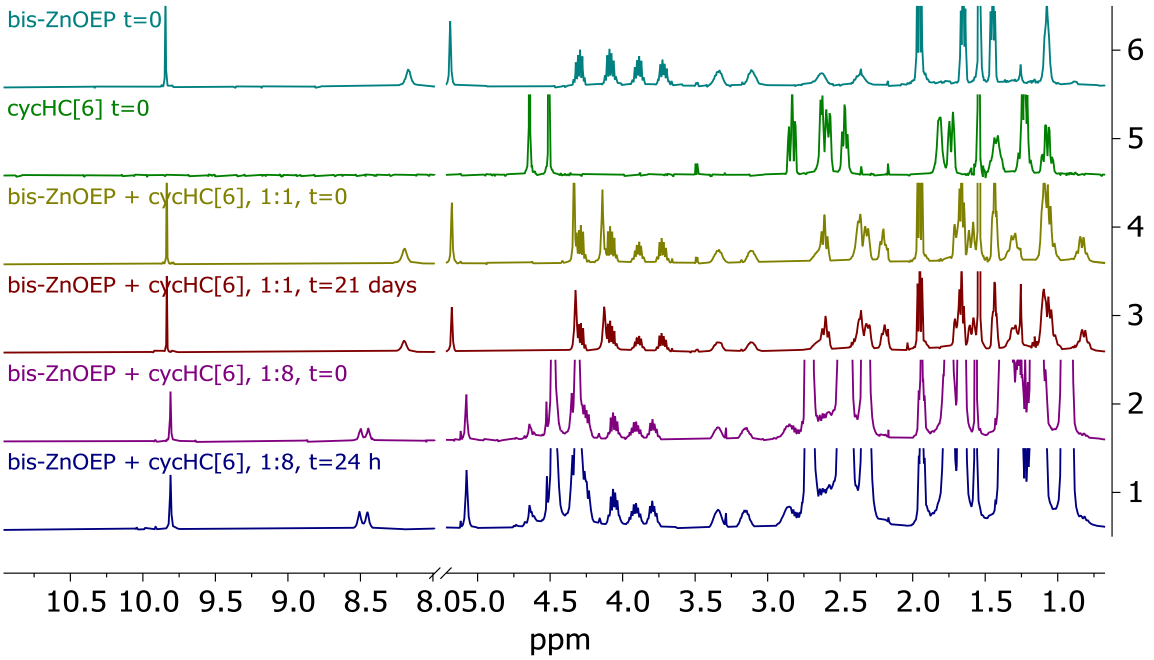

Figure S33 Spectrum of 1.0 · 10^{-3} M **bis-ZnOEP** at time zero and re-measured after 21 days; spectrum of 1.0 · 10^{-3} M (*R*,*R*)-**cycHC[6]** at time zero and re-measured after 17 days; spectrum of 1:1 ratio mixture of 1.0 · 10^{-3} M **bis-ZnOEP** and (*R*,*R*)-**cycHC[6]** measured at time zero and re-measured after 21 days. Samples were kept at room temperature. No changes in time were observed.

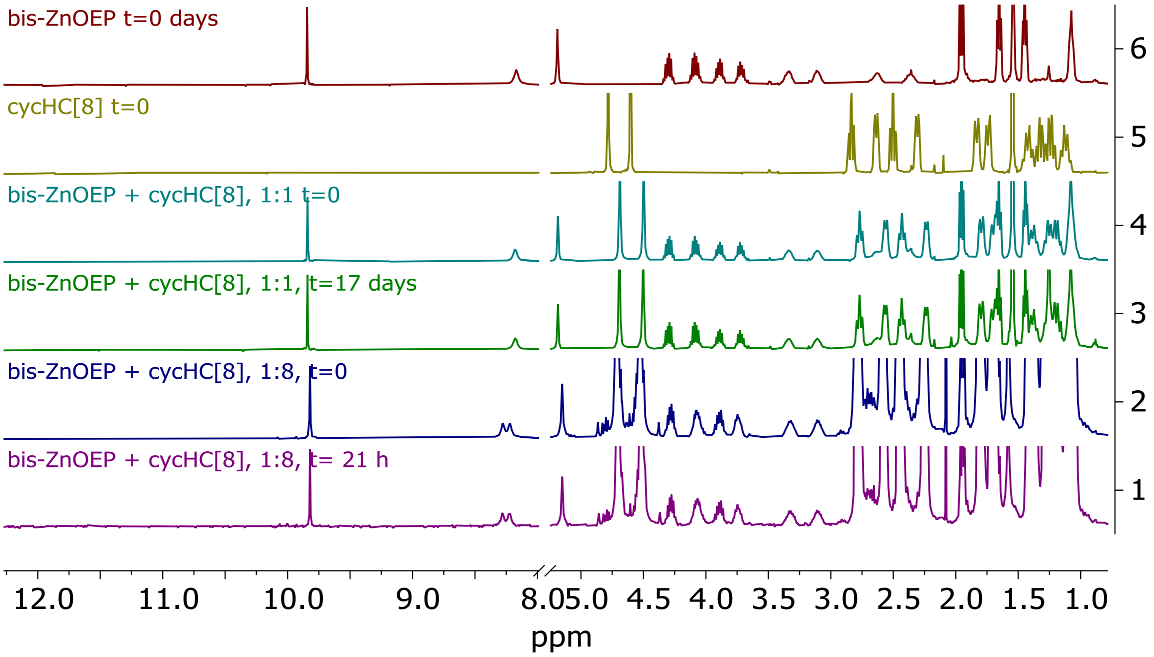

Figure S34 Spectrum of 1.0 · 10^{-3} M **bis-ZnOEP** at time zero and re-measured after 21 days; spectrum of 1.0 · 10^{-3} M (*R*,*R*)-**cycHC[8]** at time zero and re-measured after 17 days; spectrum of 1:1 ratio mixture of 1.0 · 10^{-3} M **bis-ZnOEP** and (*R*,*R*)-**cycHC[8]** measured at time zero and re-measured after 17 days. Samples were kept at room temperature. No changes in time were observed.

7. Variable temperature in ^{1}H NMR

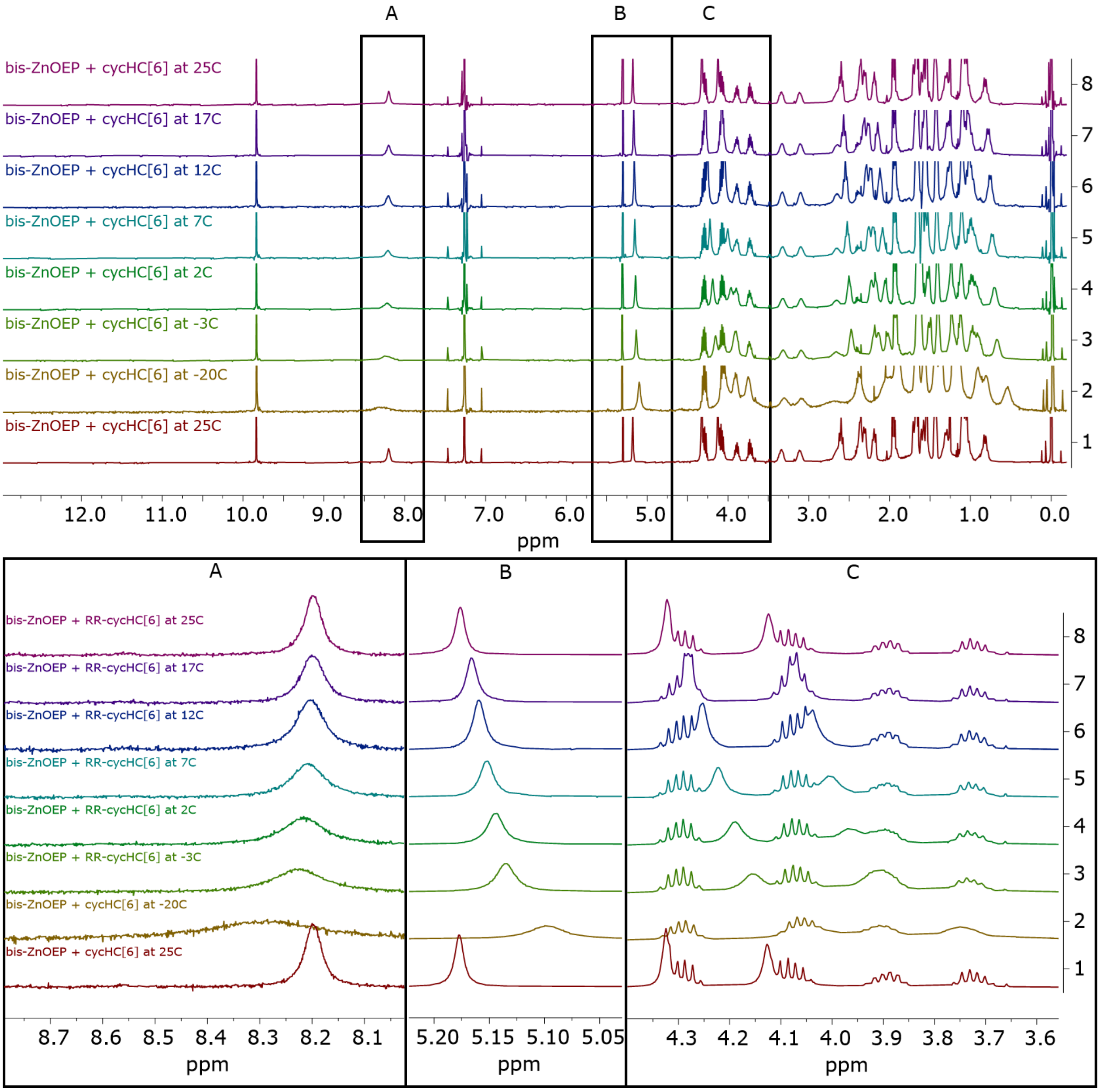

Figure S35 Spectrum of 1:1 ratio mixture of 1.0 · 10^{-3} M **bis-ZnOEP** and (*R*,*R*)-**cycHC[6]** at (from top to bottom) 298 K, 290K, 285K, 280K, 275K, 270K, 253K and 298K

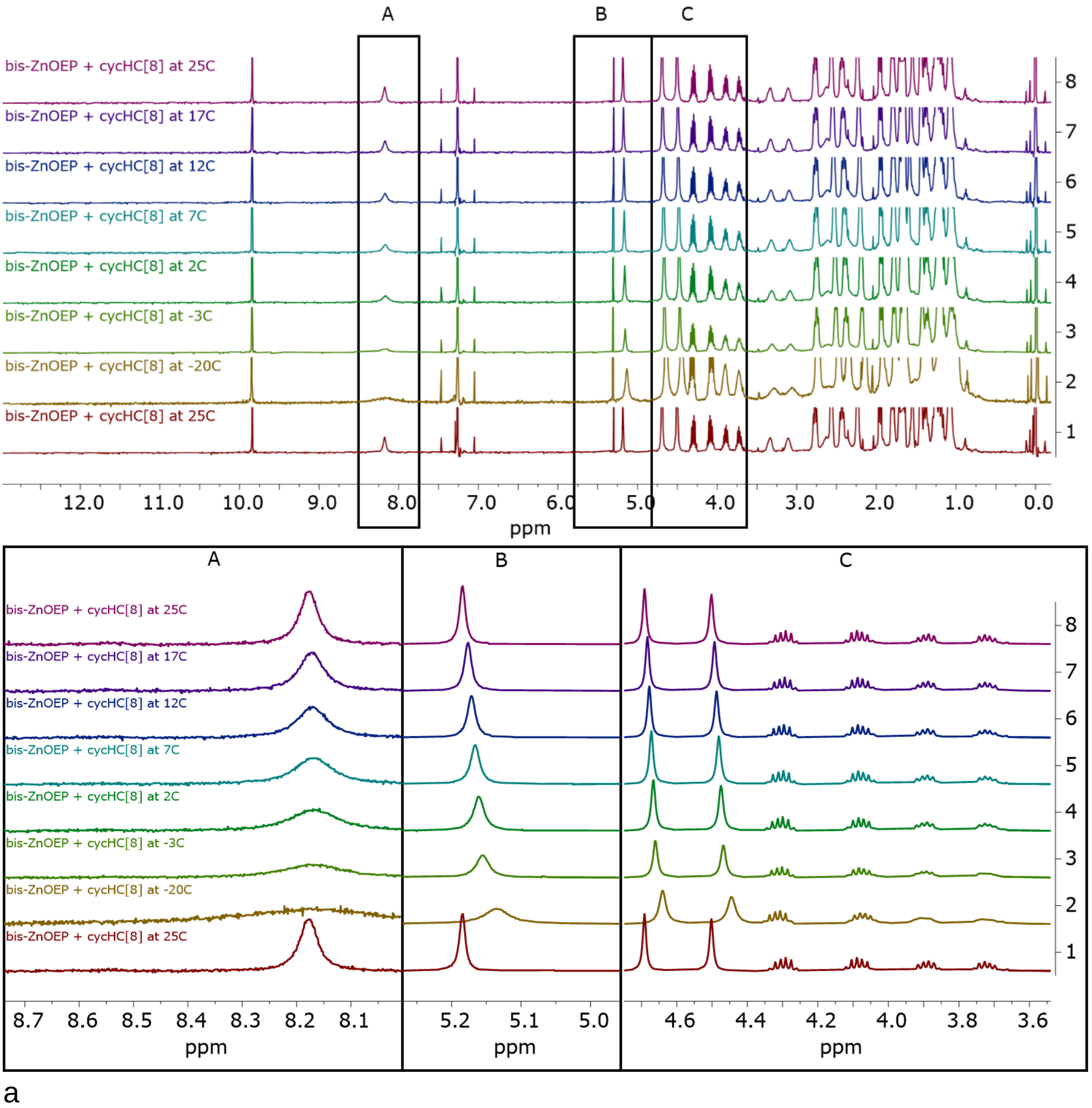

Figure S36 Spectrum of 1:1 ratio mixture of 1.0 · 10^{-3} M **bis-ZnOEP** and (*R*,*R*)-**cycHC[8]** at (from top to bottom) 298 K, 290K, 285K, 280K, 275K, 270K, 253K and 298K

8. Structural analysis of cycHC and bis–ZnOEP

We have examined previously obtained crystal structures of **bis–ZnOEP** [7–9] and of **cycHC[*n*]** with ZnTPP[10] for their similarity to herein presented complexes. All distances were measured between the centers of corresponding atoms and then the van der Waals radius were added.

Measurements for bis–ZnOEP

Measured structures covered example of pure **bis-ZnOEP** in *syn* conformation [8], complex with guanidine derivatives (1:2 stoichiometry) in *anti* conformation [9] and complex with chiral bidentate 1,2-diaminocyclohexane (1:1 stoichiometry) in tweezer–like conformation [7]. All crystals provided almost identical measures.
We measured the porphyrin core diameter (d_1) as distance of central symmetrical carbons at beta-position and wider diameter (d_2) as distance of central symmetrical carbons of methylenes from ethyl substituents (Figure S37), because they are in the same plane as the porphyrin core; all measures include van der Waals diameters of carbon atoms. Measurements were done for every porphyrin ring in all three available crystal structures.

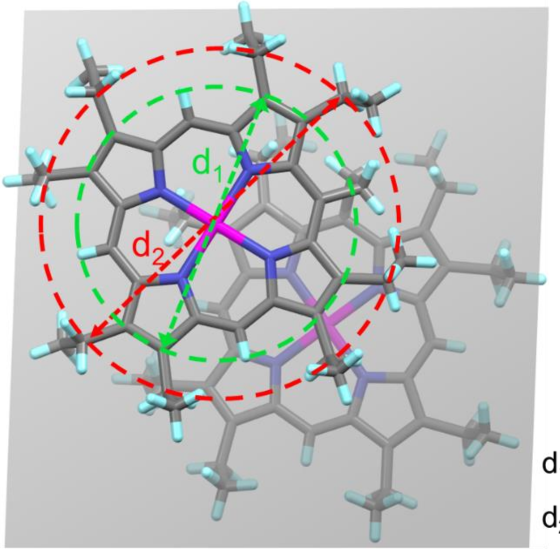

$d_1 = 12.0 \pm 0.1$ Å

$d_2 = 14.7 \pm 0.1$ Å

Figure S37 Illustration of **bis-ZnOEP** size measurements. Green circle is defined by carbons at beta position in the porphyrin ring and has diameter d_1. Red circle is defined by methylene carbons of ethyl substituents and has diameter d_2.

Measurements for cycHC

Measured structures are previously published crystals of complex between cycHC and ZnTPP.[10] We measured height and diameter; all measures of distances include van der Waals diameters of hydrogen atoms. Structures of cycHC are more flexible than **bis-ZnOEP**, therefore, the error ranges of particular measures are larger.
Height measurements: First, we defined mean equatorial plane using all six or eight methylene bridge carbons (C_{mb}) of **cycHC[6]** or **cycHC[8]**, respectively. Second, on every cyclohexanourea monomeric unit was found the hydrogen atom (H_P), which is reaching the furthest from equatorial plane. On both portals were defined mean planes by portal hydrogen atoms (H_P). Three hydrogens for particular portal of **cycHC[6]** and four hydrogens for particular portal of **cycHC[8]**. Then the height was measured as a distance of the H_P atoms from the plane of the opposite portal (Figure S38A).

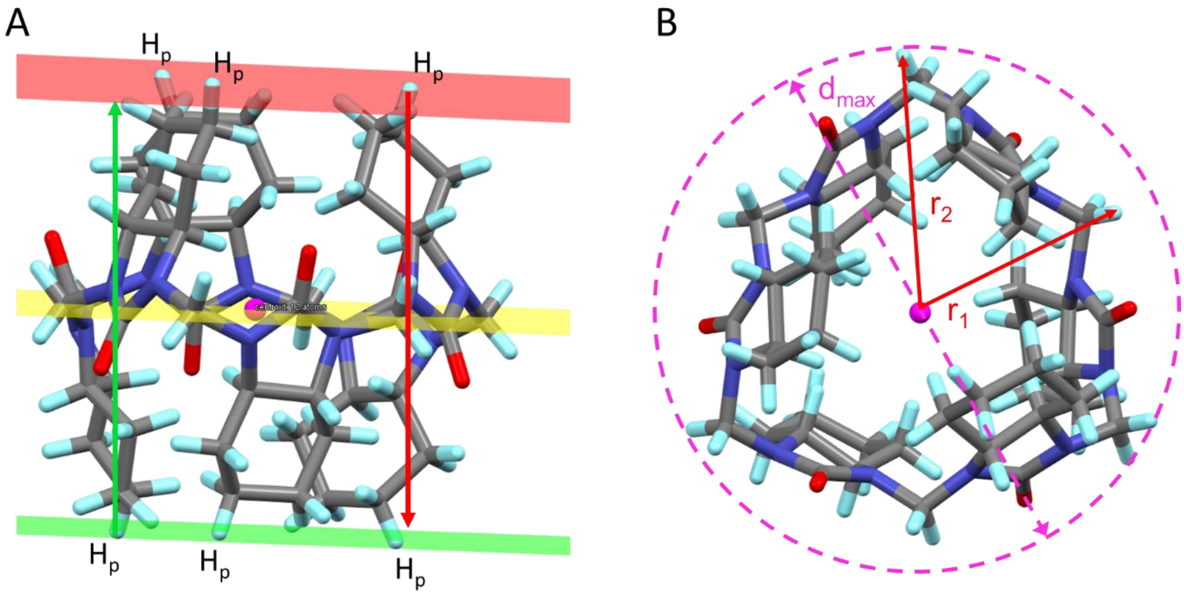

Figure S38 (A) Illustration of height measurement on **cycHC[6]**. Equatorial mean plane defined by methylene bridges is yellow and portals mean planes defined by H_p atoms are green and red. The height is distance between red plane H_p atoms and green plane; and also vice versa the distance between green plane H_p atoms and red plane. (B) Illustration of diameter measurement on **cycHC[6]**. Hydrogen atoms of methylene bridges define centroid (pink ball). Two sets of symmetrically inequivalent methylene bridges can be recognized based on their distance from centroid (r_1 and r_2)

Diameter measurements: First, we defined mean centroid using all 12 or 16 methylene bridge hydrogen (H_{mb}) atoms of **cycHC[6]** or **cycHC[8]**, respectively. Second, the distance of every H_{mb} from centroid was measured (r_1 and r_2) (Figure S38B). Results clearly showed that both cycHC has two sets of symmetrically inequivalent methylene bridges. First set of bridges is characterized by shorter distance from the centroid (r_1) and second set of bridges is characterized by longer distance from centroid (r_2). The maximum diameter (d_{max}) of cycHC is defined as $d_{max} = 2{\cdot}r_2$.

1. Borovkov, V.V.; Lintuluoto, J.M.; Inoue, Y. Synthesis of Zn-, Mn-, and Fe-Containing Mono- and Heterometallated Ethanediyl-Bridged Porphyrin Dimers. *Helv. Chim. Acta* **1999**, *82*, 919.
2. Prigorchenko, E.; Öeren, M.; Kaabel, S.; Fomitšenko, M.; Reile, I.; Järving, I.; Tamm, T.; Topić, F.; Rissanen, K.; Aav, R. Template-Controlled Synthesis of Chiral Cyclohexylhemicucurbit[8]Uril. *Chem. Commun.* **2015**, *51*, 10921–10924, doi:10.1039/C5CC04101E.
3. Aav, R.; Shmatova, E.; Reile, I.; Borissova, M.; Topić, F.; Rissanen, K. New Chiral Cyclohexylhemicucurbit[6]Uril. *Org. Lett.* **2013**, *15*, 3786–3789, doi:10.1021/ol401766a.
4. Thordarson, P. Determining Association Constants from Titration Experiments in Supramolecular Chemistry. *Chem. Soc. Rev.* **2011**, *40*, 1305–1323, doi:10.1039/C0CS00062K.
5. Brynn Hibbert, D.; Thordarson, P. The Death of the Job Plot, Transparency, Open Science and Online Tools, Uncertainty Estimation Methods and Other Developments in Supramolecular Chemistry Data Analysis. *Chem. Commun.* **2016**, *52*, 12792–12805, doi:10.1039/C6CC03888C.
6. Fomitšenko, M.; Peterson, A.; Reile, I.; Cong, H.; Kaabel, S.; Prigorchenko, E.; Järving, I.; Aav, R. A Quantitative Method for Analysis of Mixtures of Homologues and Stereoisomers of Hemicucurbiturils That Allows Us to Follow Their Formation and Stability. *New J. Chem.* **2017**, *41*, 2490–2497, doi:10.1039/C6NJ03050E.
7. Borovkov, V.V.; Fujii, I.; Muranaka, A.; Hembury, G.A.; Tanaka, T.; Ceulemans, A.; Kobayashi, N.; Inoue, Y. Rationalization of Supramolecular Chirality in a Bisporphyrin System. *Angew. Chem. Int. Ed.* **2004**, *43*, 5481–5485, doi:10.1002/anie.200460965.
8. Fujii, I.; Borovkov, V.V.; Inoue, Y. Crystal Structure of Bis-Zn-Porphyrin. *X-ray Struct. Anal. Online* **2006**, *22*, x77–x78, doi:10.2116/analscix.22.x77.
9. Osadchuk, I.; Konrad, N.; Truong, K.-N.; Rissanen, K.; Clot, E.; Aav, R.; Kananovich, D.; Borovkov, V. Supramolecular Chirogenesis in Bis-Porphyrin: Crystallographic Structure and CD Spectra for a Complex with a Chiral Guanidine Derivative. *Symmetry* **2021**, *13*, 275, doi:10.3390/sym13020275.
10. Ustrnul, L.; Kaabel, S.; Burankova, T.; Martõnova, J.; Adamson, J.; Konrad, N.; Burk, P.; Borovkov, V.; Aav, R. Supramolecular Chirogenesis in Zinc Porphyrins by Enantiopure Hemicucurbit[*n*]Urils (*n* = 6, 8). *Chem. Commun.* **2019**, *55*, 14434–14437, doi:10.1039/C9CC07150D.
